# Applying test case prioritization to software microbenchmarks

**DOI:** 10.1007/s10664-021-10037-x

**Published:** 2021-09-30

**Authors:** Christoph Laaber, Harald C. Gall, Philipp Leitner

**Affiliations:** 1 Department of Informatics, University of Zurich, Zurich, Switzerland; 2 Software Engineering Division, Chalmers | University of Gothenburg, Gothenburg, Sweden

**Keywords:** performance testing, software microbenchmarking, test case prioritization, regression testing, JMH

## Abstract

Regression testing comprises techniques which are applied during software evolution to uncover faults effectively and efficiently. While regression testing is widely studied for functional tests, performance regression testing, e.g., with software microbenchmarks, is hardly investigated. Applying test case prioritization (TCP), a regression testing technique, to software microbenchmarks may help capturing large performance regressions sooner upon new versions. This may especially be beneficial for microbenchmark suites, because they take considerably longer to execute than unit test suites. However, it is unclear whether traditional unit testing TCP techniques work equally well for software microbenchmarks. In this paper, we empirically study coverage-based TCP techniques, employing *total* and *additional* greedy strategies, applied to software microbenchmarks along multiple parameterization dimensions, leading to 54 unique technique instantiations. We find that TCP techniques have a mean *APFD-P* (average percentage of fault-detection on performance) effectiveness between 0.54 and 0.71 and are able to capture the three largest performance changes after executing 29% to 66% of the whole microbenchmark suite. Our efficiency analysis reveals that the runtime overhead of TCP varies considerably depending on the exact parameterization. The most effective technique has an overhead of 11% of the total microbenchmark suite execution time, making TCP a viable option for performance regression testing. The results demonstrate that the *total* strategy is superior to the *additional* strategy. Finally, *dynamic-coverage* techniques should be favored over *static-coverage* techniques due to their acceptable analysis overhead; however, in settings where the time for prioritzation is limited, *static-coverage* techniques provide an attractive alternative.

## Introduction

Regression testing approaches assist developers to uncover faults in new software versions, compared to previous versions. One such approach is test case prioritization (TCP): it reorders tests to execute the most important ones firsts, to find faults sooner on average. TCP has been extensively studied in unit testing research (Rothermel et al. 1999; Rothermel et al. 2001; Elbaum et al. 2001; 2002; Tonella et al. 2006; Zhang et al. 2009b; Mei et al. 2012; Yoo and Harman 2012; Zhang et al. 2013; Hao et al. 2014; Henard et al. 2016; Luo et al. 2016; Luo et al. 2018; Luo et al. 2019). The unit-testing-equivalent technique for testing performance is software microbenchmarking. However, software microbenchmarks take substantially longer to execute, often taking multiple hours or even days (Huang et al. 2014; Stefan et al. 2017; Laaber and Leitner 2018), which is a compelling reason to apply TCP to capture important performance changes sooner. Unfortunately, compared to functional regression testing, performance regression testing is not as intensively studied. So far, the focus has been on predicting the performance impact of code changes on commits to decide whether performance tests should be run at all (Huang et al. 2014; Sandoval Alcocer et al. 2016), on prioritizing microbenchmarks according to the expected performance change size (Mostafa et al. 2017), or on selecting microbenchmarks that are most likely to detect a performance regression (de Oliveira et al. 2017; Alshoaibi et al. 2019; Chen et al. 2020).

Applying traditional TCP techniques to software microbenchmarks could work well due to their similarities to unit tests, i.e., a suite contains many microbenchmarks, they are defined in code, they are self-contained and therefore rearrangeable, and they operate on a granularity-level of statements and methods. In addition, existing research builds on the assumption that traditional TCP techniques can be used as baselines for TCP on microbenchmarks (Mostafa et al. 2017). However, traditional TCP techniques might also behave differently when used to prioritize microbenchmarks, for the following reasons: (1) They rank their tests based on coverage information, under the assumption that a test covering more statements, branches, or functions is more likely to find defects. However, performance changes might not be associated with the number of covered elements, but with the performance impact of each of these elements (e.g., a change to a loop variable potentially has a bigger impact than one to multiple conditional statements (Jin et al. 2012)). (2) Where unit tests have a clearly defined binary outcome (pass or fail), software microbenchmarks result in distributions of performance counters indicating probabilistic results. (3) The reliability of software microbenchmark results and, consequently, of the performance changes is dependent on how rigorous one conducts the measurement. Hence, the effectiveness of TCP techniques could be compromised by performance measurement inaccuracies.

To investigate whether these underlying differences of unit tests and software microbenchmarks lead to measurable differences in the usefulness of existing TCP techniques, we empirically study traditional coverage-based prioritization techniques along multiple dimensions: (1) greedy prioritization strategies that rank benchmarks either by their *total* coverage or *additional* coverage that is not covered by already ranked benchmarks, (2) benchmark granularity on either *method* or *parameter* level, (3) coverage information with *method* granularity extracted either *dynamically* or *statically*, and (4) different coverage-type-specific parameterizations. In total, our study compares 54 unique TCP technique instantiations. Research has shown that the studied dimensions affect TCP effectiveness and coverage precision (Rothermel et al. 2001; Elbaum et al. 2002; Hao et al. 2014; Henard et al. 2016; Luo et al. 2016; Luo et al. 2019; Reif et al. 2016; Reif et al. 2019).

As study objects, we select 10 Java open-source software (OSS) projects with comprehensive *Java Microbenchmark Harness (JMH)* suites, having 1,829 unique microbenchmarks with 6,460 unique parameterizations across 161 versions, to which we apply all prioritization techniques.

As part of our study, we formulate and answer the three subsequent research questions:

An effective TCP technique should be able to rearrange the execution order of microbenchmarks to detect larger performance changes sooner. We investigate whether this is the case with our first research question:

**RQ 1**: *How* **effective** are TCP techniques in ranking software microbenchmarks to detect large performance changes early?

Our evaluation relies on two effectiveness metrics: (1) the average percentage of fault-detection on performance (*APFD-P*) that indicates how good a ranking is compared to an ideal ranking, with values ranging from 0 (worst) to 1 (best); and (2) the percentage of benchmarks in a suite that must be executed to find the 3 largest performance changes (*Top-3*).

We find that the best techniques achieve mean *APFD-P* values between 0.54 and 0.71 and mean *Top-3* values between 29% and 66%, depending on the project. Techniques using the *total* strategy outperform the ones with the *additional* strategy, and *dynamic-coverage* enables more effective techniques compared to *static-coverage*. Although there is a minor discrepancy in the ranking of the different techniques when considering either *APFD-P* or *Top-3*, the overall best *dynamic-coverage* and *static-coverage* techniques are consistent. We further find that all TCP techniques perform better than a *random* ranking. However, “wrong” parameterization can have detrimental effects on their effectiveness, even rendering some techniques inferior to *random* for some projects. Hence, choosing good parameter values is paramount for effectiveness.

With the second research question, we investigate the robustness of the effectiveness measures from RQ 1 when considering different magnitudes of performance changes (i.e., the difference in execution time between two versions) as significant:

**RQ 2**: *How* **robust** are the TCP techniques’ effectiveness with respect to performance change sizes?

We find that the size at which a performance change is considered significant impacts the effectiveness of TCP techniques. Depending on the technique and the project, our results show that *APFD-P* values differ between a median of 0.11 to 0.28, with a maximum of up to 0.62. However, the ranking of techniques, i.e., which techniques perform better or worse, is hardly impacted.

When considering the practical usefulness of TCP techniques, it is crucial to not only consider their effectiveness, but also how much overhead the required analysis adds to the overall benchmarking time. We define this as the efficiency of a technique and investigate this in our third research question:

**RQ 3**: *How* **efficient** are the TCP techniques?

We find that the runtime overhead of the studied techniques ranges between < 1% and 59% of the total microbenchmark suite execution duration. Techniques with *dynamic-coverage* add between 10% and 17%, and techniques with *static-coverage* often add less than 4% overhead. However, similar to our effectiveness results, choosing the “wrong” prioritization parameters for *static-coverage* techniques can result in excessive overheads even beyond 55%. This indicates that if “good” parameters are chosen, applying TCP can be highly worthwhile.

### Recommendations

In typical TCP scenarios, where the entire microbenchmark suite is executed, we suggest employing dynamic TCP techniques due to the low overhead of 11%. However, if TCP is applied in settings with strict time limits, e.g., as part of a continuous integration (CI) pipeline, the analysis overhead introduced by TCP might still exceed the available time budget. In these cases, static TCP techniques can be a viable alternative if the “right” parameters are selected. Finally, according to our results, the *total* strategy is superior to the *additional* strategy, which may be surprising to readers accustomed to similar research on unit testing, e.g., Luo et al. (2019).

### Contributions

The main contributions of our study are:

- A first large-scale empirical comparison of TCP techniques applied to software microbenchmarks, which can serve as a reference point for future research to decide which techniques and parameters to choose as baselines.
- Empirical evidence about the impact of performance change sizes and coverage-type-specific parameters on TCP effectiveness and efficiency.
- A method to conduct studies about TCP for software microbenchmarks (and, potentially, other types of performance tests).
- An extensive *JMH* microbenchmark result data set, executed in a controlled, bare-metal environment, for 10 Java OSS projects having 1,829 distinct microbenchmarks with 6,460 distinct parameterizations across 161 versions. The data set consists of 46,978,627,870 microbenchmark invocation measurements. The data set is available as part of our replication package (Laaber et al. 2021b).

## Software Microbenchmarking with *JMH*

Software microbenchmarking is a performance testing technique that measures certain performance metrics, such as execution time, throughput, or memory utilization, of small code units. These small code units are usually individual methods or statements, which makes software microbenchmarking comparable to unit tests in functional testing. In the remainder of the paper, we use both benchmark and microbenchmark to refer to software microbenchmarks.

In the Java world, *JMH* is the de facto standard framework for defining and executing software benchmarks. Similarly to *JUnit*, a benchmark is defined as Java source code with annotations. Listing 1 shows an example from *RxJava*. A benchmark is a public method annotated with @Benchmark, here measuring the performance of a latched observer (lines 8–15). *JMH* supports parameterization of benchmarks, i.e., executing the same benchmark method with multiple inputs. Parameters for benchmarks are instance variables annotated with @Param (lines 19–20), defined in a state object (@State). This state object can either be the benchmark class itself or, as in this case, a different class which is passed to the benchmark method as a parameter. In this example, the values of parameter size are 1 and 1000, resulting in the benchmark to be executed twice, once for each value. If multiple parameters are defined, the number of executions is the cross-product of their number of values.

**Listing 1:**
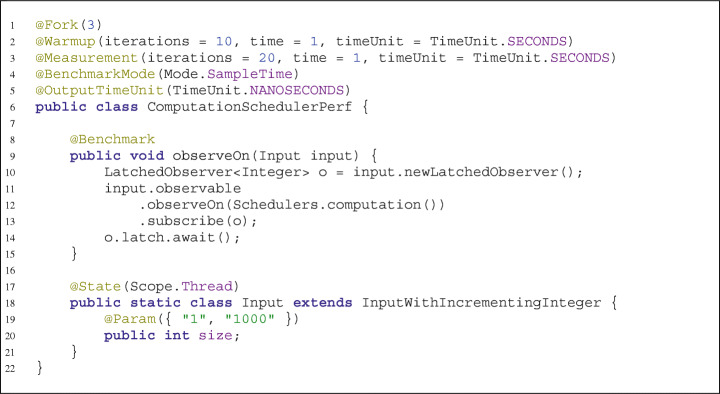
Modified *JMH* example from *RxJava*

**Fig. 1:**
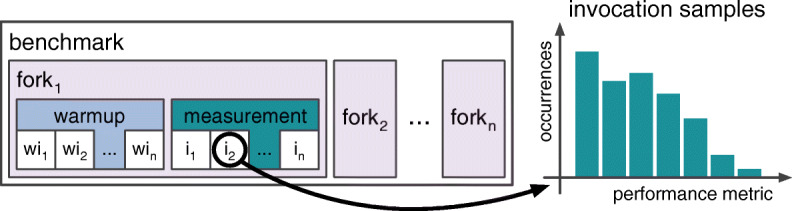
*JMH* execution

As performance is affected by multiple factors, such as the execution environment (e.g., bare-metal server, cloud, developer laptop) or the programming language (e.g., compiler optimizations, caches), one has to execute benchmarks multiple times to get reliable results. *JMH* lets developers configure the execution repetitions (lines 1–3) as well as the measured performance metric (lines 4–5). Figure 1 visualizes how *JMH* executes benchmarks (we refer to elements of the figure in “quotes”). A (parameterized) benchmark is repeatedly invoked for a defined time period (e.g., 1s), called an iteration, and the performance metric is reported. This performance metric can be the average execution time (AverageTime), the throughput (Throughput) across all invocations, or a sample distribution of the invocation values (SampleTime). *JMH* runs multiple iterations (line 2 and “warmup”) to bring the system into a steady-state, which is required for reliable measurements, followed by multiple measurement iterations (line 3 and “measurement”). To deal with non-determinism of the Java Virtual Machine (JVM) (e.g., dynamic compilation), *JMH* supports forks (line 1 and “fork”) that execute the same benchmarks in fresh JVMs. The result of a benchmark is then the distribution of results from all measurement iterations (“i”) of all forks (“fork”).

## Test Case Prioritization on Microbenchmarks

Test case prioritization (TCP) describes a set of techniques that make the regression testing effort in software evolution, i.e., when new versions are submitted for testing, more effective. The idea is to reorder the execution sequence of individual test cases in a test suite, such that tests that are executed earlier have a higher potential of exposing important faults than tests that are executed later. TCP has been extensively studied for functional unit tests (Yoo and Harman 2012), but there is only one work, to the best of our knowledge, which applies TCP to performance tests, i.e., Mostafa et al. (2017).

**Fig. 2:**
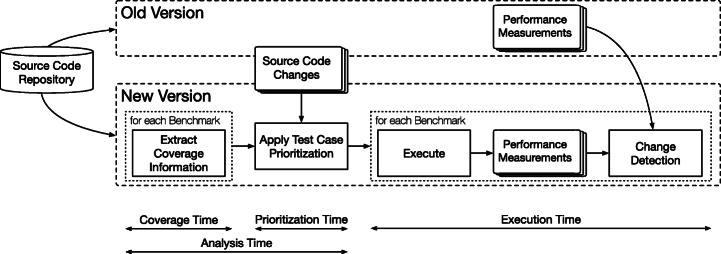
Test case prioritization (TCP) on software benchmarks approach

As microbenchmarks are different from unit tests, TCP on them also requires some adaptation. Figure 2 shows a simplified view on how we define TCP on microbenchmarks.

In this paper, we focus on the most traditional TCP techniques from unit testing research (Rothermel et al. 1999), which rely on coverage information for prioritization. Therefore, the first step upon a new version is to extract coverage information for each benchmark. This information can be on different granularity levels, such as class, method, or statement and can be extracted dynamically or statically. Note that in TCP for unit testing, dynamic coverage is extracted during the regular test execution and the information of the old version is used for prioritization of the new version. As extracting coverage during the measurement phase of a benchmark would distort the measurement result, we need to extract it in a separate phase. This phase is at the beginning of a new version, where we invoke each benchmark once with the coverage extractor injected. The time required to extract coverage information for all benchmarks is called “coverage time”.

Based on the extracted coverage information, the next step is to apply TCP to get an execution order for the benchmark suite. A dedicated TCP strategy decides how to assign each benchmark a rank based on its coverage information. The two strategies considered in this paper are the *total* and *additional* strategies, which are both greedy heuristics. The *total* strategy ranks benchmarks by their coverage set size in descending order. Benchmarks that cover more elements, e.g., methods or statements, are executed earlier than ones that cover fewer elements. The *additional* strategy iteratively selects benchmarks that have the largest coverage sets that have not been covered by an already ranked benchmark. In addition, our TCP techniques also consider the code change between the old and the new version. They perform the ranking based on the coverage information and the strategy and then split the ranked benchmarks into two sets, i.e., the ones that are affected and the ones that are unaffected by the code change. The affected ones are executed before the unaffected ones according to the ranking. Another change-aware strategy would be to only consider coverage information that has changed. However, in our experiments this did not lead to better results and, hence, we do not report these results but leave it to future work to investigate different change-aware approaches. The time required to produce the benchmark ranking is called “prioritization time”, and the sum of coverage and prioritization time is the “analysis time”.

Based on the benchmark ranking, each benchmark is then executed and their measurements are compared to the measurements of the same benchmark in the old version. A change detection procedure decides whether there has been a performance change, i.e., regression or improvement, and the developers are notified. The time required to execute the full benchmark suite is called “execution time”.

The main goal of TCP is to capture important performance changes as early as possible. In this paper, we consider the change size as the importance criterion, i.e., larger changes are more important than smaller changes. Section 6.6 discusses this aspect in more detail. To evaluate a certain TCP technique, we compare its ranking to an ideal ranking using standardized metrics and investigate whether the analysis time is reasonably small compared to the execution time. Especially coverage extraction is known to be expensive. If the analysis time is too expensive, the benefits of earlier performance change detection might not outweigh the temporal overhead compared to just running the benchmarks in random order.

## Empirical Study

To investigate whether TCP techniques originating from unit testing research are applicable to software microbenchmarks, we conduct a laboratory experiment (Stol and Fitzgerald 2018) on open-source Java projects with *JMH* software microbenchmark suites. The study compares the effectiveness and efficiency (i.e., dependent variables) of different TCP techniques, exploring a wide variety of parameter combinations (i.e., independent variables).

### Experiment Process

**Fig. 3:**
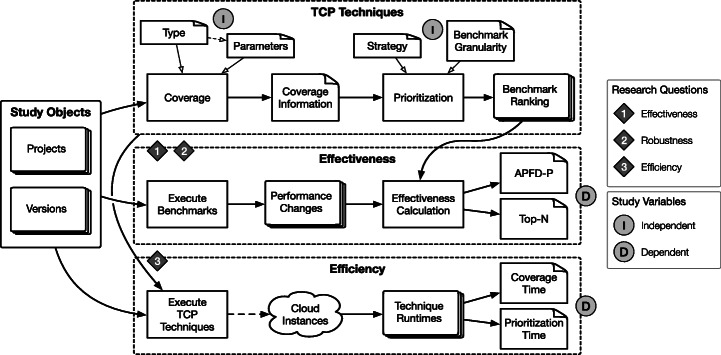
Experiment process overview

We use the research design depicted in Fig. 3. First, we select suitable projects in multiple versions as study objects (see Section 4.2). Second, for all selected versions of all selected projects, we apply the TCP techniques under study by retrieving coverage information of all benchmarks that serve as input to the actual prioritization. The parameter space, i.e., independent variables of our study  (see Section 4.3), consists of the prioritization strategy, the benchmark granularity, the coverage type, and the coverage-type-specific parameters. The result of the prioritization strategies is then an ordered list of benchmark with ranks, in descending order. Third, we compare these rankings by their effectiveness  and efficiency , which are defined by the dependent variables of our study  (see Section 4.4). For effectiveness, we execute all benchmarks of all projects in all versions (see Section 4.5), compute the performance changes between adjacent versions, and calculate their effectiveness measures with the benchmark ranking and the performance changes (see Section 4.4.1). Regarding efficiency, we execute all prioritization techniques for all projects and versions on cloud instances to assess their runtime (see Section 4.5), which consists of the time required for retrieving coverage information and prioritizing the benchmarks (see Section 4.4.2).

### Study Objects

To study TCP for software microbenchmarks, we select 10 OSS Java libraries. Because of the time-intensive nature of rigorously executing benchmarks, it is infeasible to conduct a study as ours on, for example, all projects that have *JMH* suites. Therefore, we aim to select a diverse set of projects from different domains, with varying benchmark suite sizes, and a multitude of versions to apply TCP on. To this end, we perform purposive sampling (Baltes and Ralph 2020) of Github projects based on a list of 1,545 projects with *JMH* suites from Laaber et al. (2020).

First, we apply the following inclusion criteria to each project: (1) it is the main project and not a fork, (2) the repository is available on GitHub, and (3) it has 30 benchmarks or more in the newest version. After applying the inclusion criteria, the list contains 111 projects which we arrange in descending order by their number of GitHub stars, forks, and watchers, as well as their benchmark suite size. The scripts to retrieve this list are part of our replication package (Laaber et al. 2021b).

We then manually iterate through the project list from top to bottom, giving preference to “more popular” projects with many benchmarks, and apply the following inclusion criteria until we reach 10 projects: (1) either *Maven* or *gradle* is used as build tool, (2) 10 versions or more are available as *git* tags, and (3) 10 versions or more can be compiled. Depending on the number of available, compilable versions per project and the runtime of the benchmark suites, we choose at least 10 versions covering a wide variety from multiple major, minor, and patch versions.

Table 1 depicts the final set of projects used as study objects. Our data set consists of 161 versions (“Versions”) across the 10 projects, as well as 1,829 distinct and 17,464 total benchmarks (“Benchmark Methods”) and 6,460 distinct and 59,164 total benchmark parameterizations (“Benchmark Parameterizations”) across all projects and versions. The distinct number counts each benchmark or parameterization once for all versions, whereas the total number counts these once for each occurrence in a version.

**Table 1:** Study objects

Project	Github	Domain	Versions	Benchmark		Benchmark		Runtime [h]	
				Methods		Parameterizations			
				mean	stdev	mean	stdev	mean	stdev
*Byte Buddy*	https://github.com/raphw/byte-buddy	Bytecode manipulation	31	30.74	± 8	30.74	± 8	0.26	± 0.069
*Eclipse Collections*	https://github.com/eclipse/eclipse-collections	Collections library	10	471.40	± 13	2,371.40	± 13	38.45	± 0.124
*JCTools*	https://github.com/JCTools/JCTools	Concurrent data structures	11	41.45	± 20	126.91	± 52	1.15	± 0.481
*Jenetics*	https://github.com/jenetics/jenetics	Genetic algorithms	21	49.24	± 6	49.24	± 6	0.42	± 0.053
*Log4j 2*	https://github.com/apache/logging-log4j2	Logging utilities	15	252.33	± 100	309.53	± 162	2.71	± 1.398
*Netty*	https://github.com/netty/netty	Asynchronous network communication	10	123.90	± 46	746.50	± 522	6.56	± 4.625
*Okio*	https://github.com/square/okio	Data type access, storage, and processing	11	30.64	± 3	181.64	± 20	1.56	± 0.170
*RxJava*	https://github.com/ReactiveX/RxJava	Asynchronous programming	19	157.63	± 44	842.63	± 228	7.81	± 2.113
*Xodus*	https://github.com/JetBrains/xodus	Embedded, schema-less database (DB)	11	67.00	± 10	67.00	± 10	1.33	± 0.104
*Zipkin*	https://github.com/openzipkin/zipkin	Distributed tracing system	22	55.18	± 11	55.18	± 11	0.48	± 0.101

**Fig. 4:**
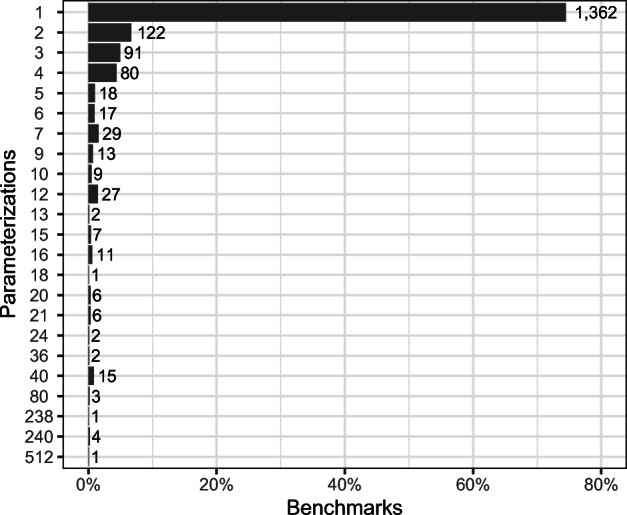
Number of parameterizations per benchmark method

The difference between “Benchmark Methods” and “Benchmark Parameterizations” is that the former considers methods annotated with @Benchmark, and the latter considers each benchmark parameterization (see Section 2) as a distinct benchmark. For both, the table reports the mean and standard deviation across a project’s versions. Note that the number of benchmarks and benchmark parameterizations is not constant across the projects’ versions; usually earlier version contain fewer benchmarks than later versions. This can be observed by the standard deviations not being 0. The mean number of benchmarks ranges from 30.74 for *Byte Buddy* to 471.40 for *Eclipse Collections*, and the mean number of benchmark parameterizations is between 30.74 for *Byte Buddy* and 2,371.40 for *Eclipse Collections*. If the number of parameterizations is equal to the number of benchmarks, the project does not make use of benchmark parameterization, i.e., *Byte Buddy*, *Jenetics*, *Xodus*, and *Zipkin*. Figure 4 depicts the number of distinct parameterizations per benchmark method. 74% of the benchmark methods have a single parameterization (or do not make use of *JMH* parameters), another 22% have between 2 and 12 parameterizations. A few individual benchmarks have extreme numbers of parameterizations, up to 512.

**Fig. 5:**
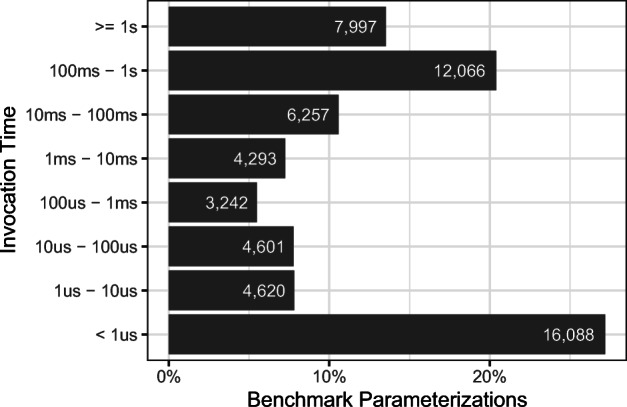
Benchmark parameterizations’ invocation times

The mean runtime (“Runtime mean”) across the versions of our projects and the execution trials (see Section 4.5) varies from approximately 16 minutes (0.26h) for *Byte Buddy* to 38.45 hours for *Eclipse Collections* for a single, full benchmark suite execution. A larger standard deviation of the runtime (“Runtime stdev”) is due to earlier versions of the respective project containing fewer benchmarks, with more benchmarks being added over time. Figure 5 shows the invocations times (x-axis), i.e., the time it takes to invoke the benchmark method once, of all benchmark parameterizations (y-axis). We observe that the invocation times are quite varied. 27% are below 1*μ* s, 48% are below 1ms, and still 14% are above 1s.

To the best of our knowledge, this is the largest data set of software microbenchmark executions across multiple versions to date. Details, including the exact versions and commit hashes used, can be found in our replication package (Laaber et al. 2021b).

### Independent Variables

Our empirical study investigates four independent variables (see Table 2): (1) the prioritization strategy, (2) the benchmark granularity, (3) the coverage type, and (4) coverage-type-specific parameters. In total, our study involves 54 unique combinations of independent variable values, thereafter simply called TCP techniques. Note that the independent variables are always concerned with how individual benchmarks are ranked, i.e., they are independent variables of the TCP techniques. We never combine performance measurements of different benchmarks or benchmark parameterizations.

**Table 2:** Independent variables and their values

Name	Short	Value	Code
	Name		Name
Prioritization Strategy	*strategy*	*total*	t
		*additional*	a
		*random*	r
Benchmark Granularity	*bench*	*benchmark-method*	m
		*benchmark-parameter*	p
Coverage Type	*cov-type*	*dynamic-coverage*	d
		*static-coverage*	s
*dynamic-coverage*:			
Benchmark Granularity	*dc-bench*	method	m
		parameter	p
*static-coverage*:			
Algorithm	*sc-algo*	RTA	R
		OCFA	0
		01CFA	01
Reflection Option	*sc-ro*	NONE	N
		OFTCAGM^1^	O
		FULL	F
		MAX^2^	M
Entry Points	*sc-ep*	single	s
		multiple	m

Variable values are listed top to bottom in increasing precision. Code names will be used as abbreviations in figures

^1^ OFTCAGM corresponds to *WALA*’s reflection option ONE_FLOW_TO_CASTS_APPLICATION_GET_METHOD

^2^ MAX represents the “best” reflection option for a particular project where the execution was successful, i.e., OFTCAGM or FULL

#### Prioritization Strategy

We study and evaluate the two most common and basic strategies from unit testing research, i.e., the *total* and *additional* strategies (Rothermel et al. 1999). The *total* strategy orders benchmarks based on the number of code elements covered by the individual benchmarks, while the *additional* strategy ranks the benchmarks based on the number of code elements that have not been covered by other, already ranked benchmarks. In addition, we compare the two strategies to a baseline with *random* benchmark order, which corresponds to the dependent variable’s mean across 100 random orderings.

#### Benchmark Granularity

Unit testing research often considers the test case granularity as an independent variable (Hao et al. 2014), which is either on test class or test method level. Since *JUnit 5*^1^ (released September 10, 2017) developers can specify parameterized test cases^2^, which arguably would be a third test granularity to consider. However, at the time of writing we are not aware of any studies that investigate TCP with test parameter granularity. *JMH* supports parameterized benchmarks since version 0.4^3^ (released February 19, 2014), and many projects make extensive use of it (Laaber et al. 2020). Therefore, our study investigates benchmark granularity on two levels: *benchmark-method* and *benchmark-parameter*. As an example, let us assume a benchmark suite *B* contains three benchmark methods *b*^1^, *b*^2^, and *b*^3^, all with two parameterizations *p* = 1 and *p* = 2. The benchmark suite to rank is then $B = \{ b^{1}_{p=1}, b^{1}_{p=2}, b^{2}_{p=1}, b^{2}_{p=2}, b^{3}_{p=1}, b^{3}_{p=2} \}$.

TCP with *benchmark-parameter* considers every parameterization of a benchmark method as the unit to rank, i.e., it takes the coverage information of each benchmark parameterization as input for the ranking. In our example, the following ranking is possible: $\langle b^{2}_{p=2}, b^{1}_{p=2}, b^{1}_{p=1}, b^{3}_{p=1}, b^{2}_{p=1}, b^{3}_{p=2} \rangle $. Here, benchmark parameterizations are individually ranked based on their coverage information, and an interleaved ranking of parameterizations of different benchmark methods is possible.

TCP with *benchmark-method* considers a benchmark method with all its parameter combinations as the unit to rank. That is, coverage information is acquired for a single parameterization of this benchmark method, the TCP ranking is computed for all benchmark methods, and parameterizations of a benchmark are ranked back to back (and not interleaved with parameterizations of other benchmarks) in descending order of their parameter values. The representative coverage information of the benchmark method is, in our case, the one of the parameterization that is ordered first (according to the descending order), because this is the one with the highest parameter values where coverage size is potentially highest. In our example, the following ranking is possible: $\langle b^{2}_{p=2}, b^{2}_{p=1}, b^{1}_{p=2}, b^{1}_{p=1}, b^{3}_{p=2}, b^{3}_{p=1} \rangle $. Here, coverage information is retrieved for $b^{1}_{p=2}$ for *b*^1^, $b^{2}_{p=2}$ for *b*^2^, and $b^{3}_{p=2}$ for *b*^3^. Note that *benchmark-method* performs the ranking on benchmark methods but executes all benchmark parameterizations; it never merges performance measurements of different benchmarks or benchmark parameterizations.

#### Coverage Type

The TCP strategies studied, i.e., *total* and *additional*, rank the benchmarks based on structural coverage information. This structural coverage can be obtained in two ways: *dynamic-coverage* and *static-coverage*. We investigate both coverage types in our study. Apart from the coverage type, the granularity it is extracted on may influence TCP effectiveness and efficiency. Different coverage granularities have been studied for unit tests such as on *statement-level* or *method-level* (Elbaum et al. 2002). In our study, we investigate *method-level* coverage granularity for two reasons: (1) *method-level* is available for both dynamic and static types; and (2) *dynamic-coverage* on *statement-level* is known to have high runtime overhead, which may render these techniques too expensive in high code velocity environments or as part of CI (Elbaum et al. 2014; Liang et al. 2018). *static-coverage* is retrieved by static call graph (CG) analyses with *WALA*, and *dynamic-coverage* is retrieved by executing a single benchmark invocation using the *JaCoCo* agent (see Section 3).

#### Coverage-Type-Specific Parameters

Previous research on TCP for unit tests investigated different prioritization strategies, coverage types and granularities, and test case granularities (Elbaum et al. 2002; Zhang et al. 2009b; Mei et al. 2012; Yoo and Harman 2012; Luo et al. 2016; Luo et al. 2019), but to the best of our knowledge, no study exists that shows the impact of different coverage-type-specific parameters on TCP effectiveness and efficiency. Coverage-type-specific parameters guide how coverage information is retrieved by their algorithms. Depending on whether *dynamic-coverage* or *static-coverage* is used, different parameters are available.

We consider the benchmark granularity (similar to benchmark granularity of the prioritization strategy) of the coverage type, i.e., of the underlying CG type, gathering the coverage information. For *dynamic-coverage*, we study method (*dc*-*bench*^*m*^) and parameter (*dc*-*bench*^*p*^) granularity, whereas for *static-coverage* we only study method granularity, as parameter granularity requires executing the benchmark or utilizing symbolic execution techniques. *dc*-*bench*^*m*^ retrieves coverage information for a benchmark method by executing a single parameterization, and *dc*-*bench*^*p*^ extracts coverage information for each benchmark parameterization.

The coverage type parameters for *static-coverage* are related to how *WALA*, a state-of-the-art static analysis library, builds the static CGs: (1) the CG algorithm (*sc-algo*), (2) the CG algorithm’s reflection option (*sc-ro*), and (3) the set of CG entry points for each benchmark (*sc-ep*).

We investigate three of the four pre-defined CG algorithms in *WALA*, the context-sensitive algorithms RTA (Bacon and Sweeney 1996) and OCFA (Shivers 1988) as well as the context-insensitive algorithm 01CFA (Grove and Chambers 2001). We refrain from using 1CFA (as an instance of nCFA), as it was shown to be inferior to both OCFA and 01CFA (Reif et al. 2019). We further exclude 01CFAContainer due to its long execution times and heavy resource usage, which led to timeouts and failures during our experiments.

Regarding the CG algorithms’ reflection options (*sc-ro*), we study all CG algorithms with no special handling of reflection (*sc-ro*^NONE^) and the highest reflection option per project that did not lead to timeouts or failures (*sc-ro*^FULL^ or *sc-ro*^OFTCAGM^). We are able to execute *Byte Buddy*, *Eclipse Collections*, *JCTools*, *Jenetics*, *Netty*, *Okio*, and *RxJava* with the highest available reflection option FULL; and *Log4j 2*, *Xodus*, and *Zipkin* with the second-highest reflection option OFTCAGM. Table 2 also lists MAX which corresponds to either FULL or OFTCAGM, depending on the project. We use MAX throughout the paper whenever we discuss a TCP technique using the highest reflection option across all projects.

Finally, CG algorithms rely on a defined set of entry points that inform the algorithm which paths of a program are executed, which classes are instantiated, and, hence, which subtypes are considered by points-to analyses. Employing different entry point sets results in different CGs and, consequently, in potentially different prioritizations (Reif et al. 2016). We construct entry point sets assuming closed-package usage, i.e., only methods that are called by the benchmark itself (@Benchmark) and setup methods (@Setup) are contained. Our study investigates two types of entry point sets: single (*sc-ep*^*s*^) and multiple (*sc-ep*^*m*^). *sc-ep*^*s*^ constructs a single entry point set for all benchmarks in a suite and, hence, builds a single CG for all benchmarks. *sc-ep*^*m*^ constructs one entry point set *per* benchmark, consisting only of the benchmark itself and its setup method(s).

### Dependent Variables

Our study investigates three types of dependent variables, two measuring TCP effectiveness and one assessing efficiency.

#### Effectiveness

For RQ 1 and RQ 2, we study two dependent variables, similar to the work by Mostafa et al. (2017): (1) average percentage of fault-detection on performance (*APFD-P*) and (2) *Top-N* percentile. These two metrics assess how effective the studied TCP techniques are in ranking benchmarks. A more effective TCP technique ranks benchmarks that uncover larger performance changes higher than benchmarks that find smaller or no performance changes. Section 6.6 discusses this and alternative definitions of TCP effectiveness as well as what an important performance change really is. We do not use *nDCG* as an effectiveness measure, as Mostafa et al. (2017) did, because *APFD-P* and *nDCG* metrics are correlated in our study.

##### Performance Changes

The performance changes detected by benchmarks between two adjacent versions are integral to the calculation of the effectiveness measures. The change size is defined as the runtime difference in percent between the previous version and the new version of the same benchmark.

**Fig. 6:**
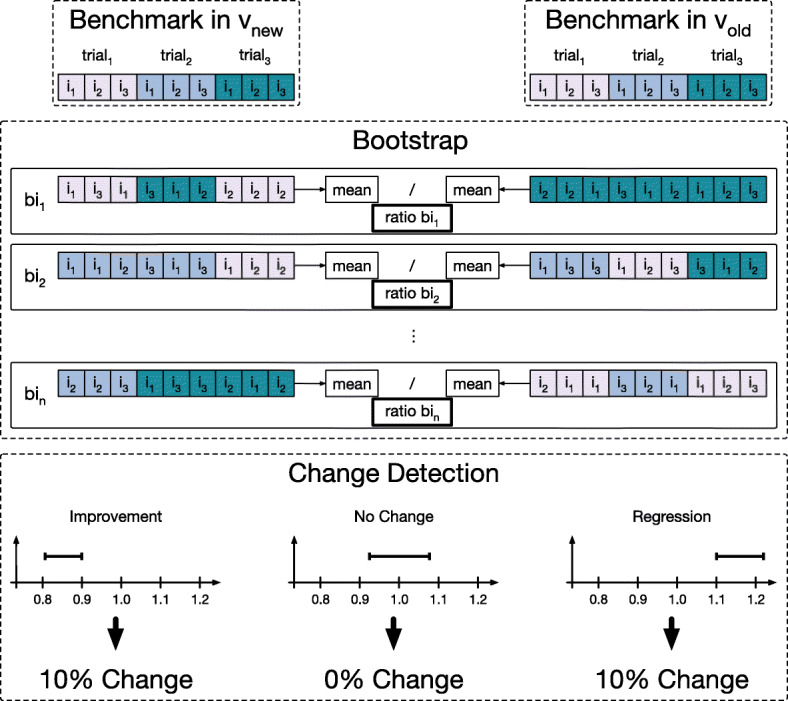
Performance change detection procedure with bootstrap confidence intervals

Rigorously assessing the change size is paramount to the internal validity of our study. Mostafa et al. (2017) use the mean runtime difference of a benchmark between two versions, i.e., an old and a new version. This, however, can be problematic as it neglects the distribution of the performance measurements. Performance measurement results are known to often be non-normally distributed (Curtsinger and Berger 2013) (e.g., long-tailed or multi-modal), and best practice suggests using bootstrap confidence intervals instead of simple average statistics, such as the mean (Kalibera and Jones 2012; Bulej et al. 2017; Bulej et al. 2019; Stefan et al. 2017; Wang et al. 2018; He et al. 2019; Laaber et al. 2019; Laaber et al. 2020). Consequently, we update Mostafa et al. (2017)’s definition of a performance change to use bootstrap confidence intervals. We depict the procedure in Fig. 6, which creates a set of bootstrap samples of ratios of mean performance changes between the old and the new version. It then uses the bootstrap samples to estimate the confidence interval of the mean performance change and deduces the change size from it.

First, we execute all benchmarks in all versions to retrieve the required measurements for change detection. We configure the benchmark suites with 10 warmup iterations and 20 measurement iterations of 1 second each. We export the invocation samples of each iteration (using *JMH*’s benchmark mode SampleTime) and take a weighted sub-sample of at most 1,000 from it. Depending on the individual benchmark invocation time, this invocation sample might be lower. If the invocation time is below 1ms, the sub-sample will contain 1,000 invocations; otherwise the sub-sample will contain the number of invocations that were executed within the 1s iteration. In the extreme case where the invocation time exceeds 1s, *JMH* executes the benchmark exactly once per iteration, and, hence, the invocation sample is 1. Section 4.2 provides an overview of our study’s benchmark invocation times. In addition, we execute the full benchmark suites of each version for 3 trials at different points in time. We decide against using the original configurations (as set by the projects’ developers) due to their extensive execution times. Already in our configuration set up, running the benchmark suites of the projects in all versions requires 2,133.81h (89 days) for three trials (see Table 1). In contrast, the configuration set by developers of the most recent version (1.3.8) of *RxJava* takes about 124.5 hours when running a single trial, which would render our study infeasible.

Second, to compute the confidence interval for the ratio of the mean, we employ a Monte-Carlo technique described by Kalibera and Jones (2012) that relies on statistical simulation, i.e., bootstrap (Davison and Hinkley 1997), with hierarchical random resampling (Ren et al. 2010) with replacement, 10,000 bootstrap iterations (Hesterberg 2015), and a confidence level of 99%. Hierarchical resampling works as follows, and as depicted in the “Bootstrap” block of Fig. 6 and Figure 2 in Kalibera and Jones (2012, p.27ff):

1. randomly select one trial from the original benchmark execution;
2. randomly select one iteration from this trial;
3. take a weighted invocation sample from this iteration;
4. repeat (2) for as many iterations as the original trial contains; and
5. repeat (1) for as many trials as the original benchmark execution contains.

Note that the procedure uses sampling with replacement, that is the same trial and iteration can occur multiple times in the bootstrap sample. The set of bootstrap samples is then defined as *R*^*b*^ in Eq. (1), where the set elements correspond to the blocks of “ratio bi” in Fig. 6.

1 $$  R^{b} = \bigcup\limits^{bi} \frac{mean(S_{new}^{b})}{mean(S_{old}^{b})} $$

*b* corresponds to the benchmark the set was acquired for, with *bi* = 10,000 bootstrap iterations. $S_{new}^{b}$ is a bootstrap sample for benchmark *b* in the *new* version, and $S_{old}^{b}$ a sample for the previous (*old*) version of the same benchmark. Each bootstrap sample *S*^*b*^ consists of at least 60 individual measurements (3 trials × 20 measurement iterations × 1 invocation) and up to 60,000 individual measurements (3 trials × 20 measurement iterations × 1,000 invocations) in our study. *mean* is the arithmetic mean.

The confidence interval bounds of *R*^*b*^ are then defined as $r^{b}_{upper}$ for the upper and $r^{b}_{lower}$ for the lower bound in Eqs. (2) and (3), respectively.

2 $$  r^{b}_{upper} = quantile_{1-\frac{1-cl}{2}} (R^{b}) $$

3 $$  r^{b}_{lower} = quantile_{\frac{1-cl}{2}} (R^{b}) $$

*quantile* returns the n^th^ quantile, and *cl* defines the confidence interval’s confidence level. In our study, we employ *cl* = 0.99 for a 99% confidence level.

Third, we define a performance change based on *R*^*b*^ and its confidence interval bounds $r^{b}_{upper}$ and $r^{b}_{lower}$. The “Change Detection” block of Fig. 6 shows the three possible change scenarios:

**improvement**: The benchmark in the new version has a statistically significant lower runtime performance as in the old version. This can be detected if the upper bound of the confidence interval is below 1.
**no change**: The benchmark in the new version has statistically the same runtime performance as in the old version. This can be detected if the confidence interval straddles 1.
**regression (slowdown)**: The benchmark in the new version has statistically significant higher runtime performance as in the old version. This can be detected if the lower bound of the confidence interval is above 1.

Equation (4) formally defines this change definition and the corresponding change size as the function *change*(*b*).

4 $$  change(b) = \begin{cases} (1 - r^{b}_{upper}) \times 100  & \iff r^{b}_{upper} < 1 \\ 0        & \iff r^{b}_{lower} < 1 \wedge r^{b}_{upper} > 1 \\ (r^{b}_{lower} - 1) \times 100  & \iff r^{b}_{lower} > 1 \end{cases} $$

Both change cases are multiplied by 100 reflecting a change in percent, e.g., 10%. Note that going forward, we do not distinguish between improvement and regression but are only concerned with performance changes in general, similar to Mostafa et al. (2017).

Compared to Mostafa et al. (2017)’s definition of a change, ours takes the measurement variability of the benchmarks into account. It is a conservative change definition that ensures that if the experiment is repeated 100 times, the mean performance change would be at least of the size reported by *change*(*b*) in 99 cases.

Chen and Shang (2017) showed that benchmarks commonly indicate many small performance changes between version pairs, which might partially be caused by measurement inaccuracy or bias (Mytkowicz et al. 2009; Curtsinger and Berger 2013; de Oliveira et al. 2013). Many of these changes are likely to be unimportant, hence distorting effectiveness measures. In RQ 1, we only consider performance changes of 3% or larger as significant, similar to Georges et al. (2007). All changes below that threshold are discarded, i.e., set to 0. In RQ 2, we explicitly investigate the effectiveness robustness, i.e., the impact the performance change size has on the studied effectiveness measures, by performing a sensitivity analysis on this threshold value. Our study investigates thresholds *t* from 0% (i.e., all changes) to 100%, where *t* ∈{0,1,2,3,4,5,6,7,8,9,10,15,20,25,50,100}.

##### *APFD-P*

The effectiveness measure *APFD-P* is adapted from the standard TCP measure average percentage of fault-detection (APFD), which was first introduced by Rothermel et al. (1999) and has since been widely used in unit testing research (Rothermel et al. 2001; Elbaum et al. 2002; Mei et al. 2012; Zhang et al. 2009a; Hao et al. 2014; Luo et al. 2016; Luo et al. 2018). APFD is a metric to assess the fault-detection capabilities of a TCP technique. It assigns a value between 0 and 1 to a benchmark ranking, where rankings with higher APFD values detect more faults sooner than rankings with lower APFD values.

As unit tests have a binary outcome (i.e., they either pass or fail), and benchmarks have a continuous result (e.g., 10% or 50% slowdown), Mostafa et al. (2017) adapted APFD for performance tests and benchmarks to incorporate different performance fault severities (i.e., performance change sizes). *APFD-P* is defined in Eq. (5).

5 $$  APFD\text{-}P = \frac{ \sum\limits_{x = 1}^{N} \frac{ detected(x) }{ T } }{ N } $$

*N* is the benchmark suite size, *T* is the total sum of all performance changes, and *detected*(*x*) returns the cumulative performance change of the first *x* benchmarks (see Eq. (6)).

6 $$  detected(x) = \sum\limits_{i = 1}^{x} change(i) $$

*change*(*i*) is the performance change of the i^th^ benchmark in a TCP ranking, according to our adapted version from Eq. (4).

##### *Top-N*

This dependent variable provides a better intuition regarding the advantages developers have from prioritizing their benchmarks. It represents the number of benchmarks in a suite that have to be executed, based on a TCP technique’s ranking, to capture the *N* largest performance changes. Following Mostafa et al. (2017), we choose *N* = 3 in our study. This captures how large a fraction of the benchmark suite must be executed to detect the 3 largest performance changes.

#### Efficiency

Effectiveness of TCP techniques captures only one part of their quality and applicability. Techniques that rely on precise analyses might produce effective results, but may be unrealistic to perform in practice due to their long runtimes. Hence, our efficiency analysis (for RQ 3) complements the effectiveness analysis by studying the runtimes of the different TCP techniques.

The efficiency dependent variable can be split into two parts, i.e., (1) coverage time and (2) prioritization time, which together form the analysis time of a TCP technique (see Section 3). Depending on the TCP technique’s independent variable values, these two times are expected to contribute in different proportions to the analysis time. The prioritization time’s computational complexity is $\mathcal {O}(m n)$ for the *total* strategy and $\mathcal {O}(m^{2} n)$ for the *additional* strategy, where *m* is the number of benchmarks and *n* the number of called production methods (Rothermel et al. 2001). In our efficiency analysis, we are interested in the actual overhead for the studied projects, with respect to the duration of executing the entire benchmark suite.

### Execution Setup

As our empirical study relies on measuring performance, i.e., (1) the performance changes of the benchmarks for each version, which are required for the effectiveness metrics (RQ 1 and RQ 2); and (2) the efficiency analysis of RQ 3, a rigorous methodology is required to reduce validity concerns and enable replicability.

#### Performance Changes

Measuring benchmark performance requires careful experiment planning because of widely reported measurement uncertainties (Georges et al. 2007; Mytkowicz et al. 2009; Curtsinger and Berger 2013; de Oliveira et al. 2013). A sloppy measurement methodology results in unreliable benchmark results, which in turn might distort the results of our experiment. To mitigate these sources of uncertainty, we apply the following steps:

1. We manually patch the build scripts of all projects and versions with the same *JMH* version (i.e., 1.21), compile the *JMH* fat Java Archives (JARs), and execute the benchmarks with Java Development Kit (JDK) version 1.8.0_181-b13 employing Java HotSpot 64-Bit Server VM (build 25.181-b13). This way we ensure that a benchmark performance change does not stem from a JDK-related or *JMH*-related improvement or regression.
2. As performance engineering best practice suggests utilizing controlled environments, we use a non-virtualized (“bare-metal”) server hosted at the first author’s university. This server has a 12-core Intel Xeon X5670@2.93GHz central processing unit (CPU) with 70 GB memory, runs ArchLinux with a kernel version 5.2.9-arch1-1-ARCH, and uses a Samsung SSD 860 PRO SATA III disk.

#### Efficiency Analysis

For the efficiency analysis, the environment used to measure the coverage and prioritization times is hosted in the private cloud of the first author’s institution. We select private cloud instances and refrain from using a bare-metal machine for two reasons: (1) the runtime of the individually measured times, i.e., coverage and prioritization, is longer than the individual benchmarks’ runtimes. They are likely in the order of seconds to minutes. Therefore, small measurement errors are not expected to have an impact on the overall result of RQ 3; and (2) the time to run the efficiency analysis would take about 77 days for a single measurement, for all TCP techniques, projects, and versions. (1) Considering performance engineering best practice and running the measurements repeatedly (e.g., 10 trials), the total duration would exceed a sensible time frame. Hence, we measure coverage and prioritization times once on multiple private cloud instances with the same configuration to make use of experiment parallelization. All instances have the same specification:

1. The cloud instance types have 16 virtual CPUs and 62 GB memory. The CPUs’ model is Intel Xeon E3-12xx v2 (Ivy Bridge, IBRS) with 2.5 GHz and a 4 MB cache.
2. The instances are provisioned with Ubuntu 18.04 LTS and run a Linux kernel version 4.15.0-23-generic.
3. Identical to the performance change execution setup, we execute the measurements with JDK version 1.8.0_181-b13 employing Java HotSpot 64-Bit Server VM (build 25.181-b13).

### Tooling, Analysis Scripts, and Data

The tools, scripts, and data required to run (and replicate) our study consists of three parts: (1) the benchmark analysis and prioritization tool *bencher* (Laaber 2020a), (2) the performance change analysis tool *pa* (Laaber 2020b), and (3) an openly-available replication package (Laaber et al. 2021b).

*bencher* is written in Kotlin 1.3.72. It parses the byte code of *JMH* projects for their benchmarks with *ASM*^4^7.2, retrieves static coverage information with *WALA*^5^1.5.0 and dynamic coverage information with *JaCoCo*^6^0.8.5, and applies the TCP techniques.

*pa* is written in Go and implements efficient, multi-threaded performance change analysis of benchmark results, as required for Section 4.4.1 and introduced by Kalibera and Jones (2012). It computes bootstrap confidence intervals and confidence interval ratios of a specified statistic (e.g., the arithmetic mean), with hierarchical random resampling with replacement, user-defined bootstrap iterations and confidence levels, and sampling of invocations.

The replication package contains all scripts that perform data preparation and cleaning, invocation of the aforementioned tools, data analyses, and data representations, as well as all input, intermediate, and output data.

### Threats to Validity and Limitations

#### Construct Validity

We rely on *APFD-P* and *Top-3* as measures for TCP effectiveness (Mostafa et al. 2017). *APFD-P* is adapted from APFD, which, although widely used, has been discussed to have limitations (Rothermel et al. 1999). We address this threat by also investigating *Top-3*. Choosing *N* = 3, as opposed to for example 1 or 5, is based on previous research (Mostafa et al. 2017), and we manually confirmed that a larger *N* would always result in effectiveness values of close to 100% (that is, the whole benchmark suite has to be executed for capturing the top *N* performance changes). We further adapt the performance change definition from Mostafa et al. (2017) to be more robust against performance measurement variabilities. Our definition uses either the lower or upper bound of the mean performance change’s confidence interval, for slowdowns or improvements, respectively. This is a conservative definition, i.e., it reflects the smallest possible change and not, for example, the largest one, according to the estimated confidence interval. However, it also provides statistical guarantees that the performance change is at least of the detected size. This improves construct validity that our effectiveness metrics are computed correctly. Nonetheless, different effectiveness metrics or performance change definitions might lead to different effectiveness results and, consequently, to different conclusions. Finally, we combine the effectiveness findings (RQ 1, RQ 2) with an efficiency analysis (RQ 3) in our discussion to provide a more holistic evaluation of TCP.

#### Internal Validity

Valid performance changes are paramount to the study’s internal validity. Measurement uncertainty is common (Mytkowicz et al. 2009; Curtsinger and Berger 2013; de Oliveira et al. 2013) and could threaten our effectiveness (i.e., validity of *APFD-P*) and efficiency results. For the performance measurements of all versions, we follow a rigorous methodology based on state-of-the-art best practice (Georges et al. 2007) utilizing a bare-metal environment. However, measurement uncertainty can never be excluded entirely. The execution configuration contributes to the reliability of the measurements, i.e., more measurements lead to more stable results. We execute each benchmark for 3 trials consisting of 20 iterations of 1s duration. This configuration is in line with other recent performance engineering works, e.g., Blackburn et al. (2016), Chen et al. (2020), and Mühlbauer et al. (2020). Nonetheless, it does not ensure that the measurements are stable, i.e., measurement variability is low. Our statistical technique for detecting performance changes (i.e., bootstrap confidence intervals) considers the measurement distributions and should, therefore, be reasonably robust. Also running more trials, which would decrease variability and increase measurement reliability, is infeasible as executing our projects in all version already took 89 days to finish. However, due to this variability, the detected performance changes have a tendency to underestimate the real change, which can have an impact on our study’s results and conclusions. In our replication package (Laaber et al. 2021b), we show that the error rate, i.e., the difference between the detected and an artificially injected change, is low, on average 1.2% for a 100% change.

We rely on statistical simulation, i.e., bootstrap confidence interval of the ratios of the mean (Kalibera and Jones 2012), to decide whether a benchmark’s result has changed between two adjacent versions. Bootstrap is a randomized algorithm to approximate a benchmark’s result population from a measured sample. Consequently, the detected performance change size might suffer from Monte-Carlo noise. We mitigate this by following statistical best practice and using 10,000 bootstrap iterations (Hesterberg 2015).

The efficiency measurements are executed in cloud environments which might interfere with the measurement. However, because the times we measure, i.e., coverage and prioritization, are in the order of minutes (or even longer), and we compare them to the total runtime of the suites, which are between 16 minutes and 38 hours, small measurement inaccuracies are not expected to change our overall conclusions for RQ 3.

Further threats to internal validity concern potential functional (RQ 1, RQ 2) and performance (RQ 3) bugs of our tool chain. We dedicated extensive effort in unit testing our tool chain and performance benchmarking core functionality. To address validity threats regarding *WALA* usage and configuration, we rely on results and best practice of current research in static analysis (Reif et al. 2016; Reif et al. 2019).

#### External Validity

Generalizability of our study is mostly concerned with the choice of our projects and versions. We selected 10 Java OSS projects in 161 versions and with 6,460 distinct *JMH* benchmark parameterizations. Although we can not generalize our findings to all Java/*JMH* projects, the data set created for this study is, to the best of our knowledge, the most extensive microbenchmarking data set to date. More projects would have rendered our study infeasible because of the time-intensive nature of running rigorous performance experiments. We picked Java because benchmark suites written in it are long-running (Laaber and Leitner 2018; Laaber et al. 2020) and, hence, would benefit from TCP. Regarding the benchmark framework, *JMH* is the de facto standard for Java at the time of study (Stefan et al. 2017; Leitner and Bezemer 2017). We selected projects that are large, well-known, popular projects from different domains to investigate high-quality software projects. However, the results might not generalize to closed-source or industrial software, other programming languages, or even other software written in Java.

We studied a specific type of performance test, i.e., software microbenchmarks. They typically measure execution runtime of small software components, such as methods or statements. Therefore, our results may not generalize to regression testing for other performance test types, e.g., load tests or system benchmarks, or other performance metrics, e.g., memory, input/output (I/O).

Finally, depending on which static CG library is employed for coverage extraction, effectiveness and efficiency results are likely to change. We chose *WALA* because it works well for software libraries such as our projects, performs reasonably well in the presence of reflection, and has been used in previous testing research (Reif et al. 2019; Luo et al. 2016).

#### Limitations

We limited the implementation of the static CG (“S”) and dynamic coverage (“D”) extractors, which occasionally causes empty coverage sets for affected benchmarks. (1) “S” and “D”: We only consider calls to study-object-internal methods as relevant for the coverage, because we are primarily interested in ranking benchmarks higher that find performance changes in the production code of the projects. Some benchmarks test JDK collections, JDK concurrency features, or atomic data types, which serve as baselines for the benchmarks of custom functionality. We consider such benchmarks not interesting for regression testing. (2) “S”: If a benchmark implementation (annotated with @Benchmark) is located in a super-class and its parameterization (@Param) is defined in the sub-class, the static CG coverage detector is not able to capture this benchmark. (3) “D”: If a *JMH* parameter value contains a comma “,”, our tooling is not able to execute this benchmark through the *JMH* command line interface, because *JMH* 1.21 exits with a parsing error. In our study five benchmarks of *RxJava*^7^ and one benchmark of *Netty*^8^ are affected by this limitation.

## Results and Analyses

This section presents our results and analyses along the three research questions. We elaborate on the impact of different independent variable value combinations on the dependent variables, i.e., effectiveness (*APFD-P* and *Top-3*) and efficiency.

The result analyses and interpretations in this section are supported by Table 3, which provides statistics about the extracted static and dynamic coverage information. Every row corresponds to a unique combination of the coverage independent variable values, i.e., coverage type (“Coverage Type”) and coverage-type-specific parameters (“Coverage Parameters”). These results support and explain phenomena observed throughout this section. Column “Covered Methods” depicts the number of called methods from each of the 59,164 benchmark parameterizations across all versions. Column “Coverage Overlap” shows the overlap of covered methods with *another* benchmark parameterization (of the same project and version). For example, the benchmarks call on average 130.52 ± 223.09 methods (directly or indirectly) of which 37%± 31% are also covered by another benchmarks, if we retrieve *dynamic-coverage* with the parameter *dc*-*bench*^*p*^. The other columns “Empty Coverage Set” show the percentage of benchmarks for which no coverage information can be extracted. Column “all” depicts the percentage of all benchmarks, whereas “1^st^”, “2^nd^”, and “3^rd^” shows it for the top 3 benchmarks.

**Table 3:** Coverage statistics of the studied coverage parameters

Coverage Type	Coverage Parameters			Covered Methods		Coverage Overlap		Empty Coverage Set			
				mean	stdev	mean	stdev	All	1^st^	2^nd^	3^rd^
*dynamic-coverage*	parameter			130.52	± 223.09	37%	± 31%	< 1%	< 1%	< 1%	< 1%
	method			211.85	± 352.35	31%	± 35%	< 1%	< 1%	1%	2%
*static-coverage*	01CFA	MAX	multiple	106.29	± 605.36	31%	± 45%	38%	32%	28%	26%
	01CFA	MAX	single	452.95	± 979.17	31%	± 45%	34%	31%	30%	27%
	01CFA	NONE	multiple	105.84	± 603.47	30%	± 45%	38%	31%	28%	27%
	01CFA	NONE	single	445.24	± 1,005.79	30%	± 45%	33%	31%	29%	27%
	OCFA	MAX	multiple	174.94	± 787.32	26%	± 41%	29%	26%	23%	24%
	OCFA	MAX	single	664.69	± 1,378.55	26%	± 41%	28%	25%	25%	24%
	OCFA	NONE	multiple	165.77	± 719.77	25%	± 41%	30%	28%	26%	26%
	OCFA	NONE	single	563.93	± 1,134.97	25%	± 41%	29%	26%	27%	25%
	RTA	MAX	multiple	7,497.67	± 26,532.51	37%	± 45%	17%	20%	17%	18%
	RTA	MAX	single	14,855.13	± 33,358.23	37%	± 45%	17%	20%	17%	18%
	RTA	NONE	multiple	1,248.64	± 8,587.39	29%	± 41%	21%	23%	20%	19%
	RTA	NONE	single	3,005.32	± 9,828.09	29%	± 41%	18%	22%	19%	18%

The *dynamic-coverage* coverage parameter is *dc-bench*. The *static-coverage* coverage parameters are *sc-algo*, *sc-ro*, and *sc-ep* (in this order)

The interested reader can find more detailed results, figures, and tables for each project in our replication package (Laaber et al. 2021b).

### RQ 1: Effectiveness

This section presents and discusses the effectiveness measures, i.e., *APFD-P* and *Top-3*, for each project and across all projects. For this, we follow a rigorous, three-step approach for the statistical analyses, as described below:

1. We calculate the effectiveness values as described in Section 4.4.1 for every combination of the projects, their versions, and the 54 TCP techniques (i.e., unique combination of the independent variable values of our study). This results in a single effectiveness value, i.e., either *APFD-P* or *Top-3*, for each combination. Recall that we use the performance change size threshold *t* = 3.
2. We then apply the Scott-Knott effect size difference (ESD) v2 test (Tantithamthavorn et al. 2019) for every project, which clusters the TCP techniques into statistically different groups *iff* the Cohen’s d (Cohen 1992) effect size estimate is non-negligible, i.e., *d* > 0.2 at significance level *α* = 0.05. Techniques in the same cluster only have a negligible effect size difference among each other and, hence, perform statistically comparably. Colloquially, better techniques receive lower ranks than worse techniques, e.g., techniques with rank 1 are better than techniques with rank 2, and so on.
3. Finally, we apply the Scott-Knott test again —the double Scott-Knott test (Tantithamthavorn et al. 2019)— this time on the ranks from the previous step across all projects. By that, we can draw general conclusions on the effectiveness of the 54 TCP techniques across all 10 projects.

#### APFD-P

##### Per Project

Table 4 shows per project the mean *APFD-P* values across all versions and all 54 TCP techniques (“Mean”), where “Max.” and “Min.” corresponds to the mean *APFD-P* value (across all versions) of the best and worst technique, respectively. For each TCP technique, we compute a 95% confidence interval of the mean across all versions with bootstrap. Column “Conf. Int.” depicts the minimal lower bound (“Lower”) and the maximal upper bound (“Upper”) of all confidence intervals. These confidence interval bounds supply a range of *APFD-P* values per project. Finally, column “vs. random” shows the number of TCP techniques that perform statistically better (“***+***”), equal (“***=***”), or worse (“***−***”) than a *random* benchmark ordering, as assessed by the ranks of the first application of the Scott-Knott test (analysis step 2). Note that the *random* ordering achieves a mean *APFD-P* value (across 100 random orderings) of approximately 0.5.

**Table 4:** *APFD-P* of the 54 TCP techniques per project compared to a *random* ordering

Project	Mean		Conf. Int.		vs. *random*		
	Max.	Min.	Upper	Lower	***+***	***=***	***−***
*Byte Buddy*	0.64	0.43	0.69	0.37	40	8	6
*Eclipse Collections*	0.64	0.60	0.71	0.53	54	0	0
*JCTools*	0.60	0.45	0.68	0.38	4	0	50
*Jenetics*	0.62	0.49	0.70	0.40	3	27	24
*Log4j 2*	0.64	0.43	0.68	0.38	22	0	32
*Netty*	0.65	0.43	0.76	0.34	31	8	15
*Okio*	0.70	0.42	0.76	0.36	33	6	15
*RxJava*	0.59	0.48	0.64	0.43	24	10	20
*Xodus*	0.71	0.51	0.74	0.43	46	4	0
*Zipkin*	0.54	0.48	0.61	0.42	27	11	16

We observe that the mean *APFD-P* values range from 0.42 for *Okio* to 0.71 for *Xodus*, with confidence interval bounds between 0.34 for *Netty* and 0.76 for *Netty* and *Okio*. The best techniques for each project range between 0.54 (*Zipkin*) and 0.71 (*Xodus*).

Compared to a *random* ordering, it depends on the project and TCP technique whether TCP on benchmarks is more effective and, therefore, provides a benefit. We see three kinds of projects:

- the ones where the majority of the TCP techniques perform better than *random*, i.e., *Byte Buddy*, *Eclipse Collections*, *Netty*, *Okio*, and *Xodus*;
- the ones where the are a similar number of techniques that are better and worse (or equal) to *random*, i.e., *Log4j 2*, *RxJava*, and *Zipkin*; and
- the ones where the majority of techniques are inferior to *random*, i.e., *JCTools* and *Jenetics*.

This shows that for most projects, a wrong TCP technique or the wrong parameterization can have detrimental effects on its effectiveness, rendering the technique inferior to a *random* ordering. Nonetheless, for every project there exists at least a few techniques that outperform *random* substantially.

##### Overall

**Fig. 7:**
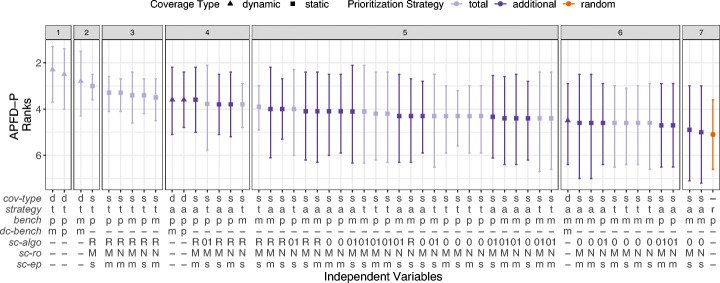
*APFD-P* ranks across all projects and version, and for a threshold *t* = 3*%*. For the independent variable descriptions, see Table 2

To assess how effective TCP on benchmarks is across all projects, we depict the results of the double Scott-Knott test (analysis step 3) in Fig. 7. The y-axis shows the *APFD-P* ranks from the first Scott-Knott test (analysis step 2), the shape represents the mean rank across the 10 projects, and the whiskers represent the 95% confidence interval of the mean computed with bootstrap. The x-axis shows the 54 TCP techniques. The facets show the ranks of the second Scott-Knott test. Techniques with the same rank (i.e., in the same facet) perform statistically comparable, and techniques with different ranks are statistically different. Colloquially, the higher on the y-axis and the more to the left on the x-axis, the better a particular TCP technique performs.

The first and most interesting observation is that the *total* strategy outperforms the *additional* strategy, with the first three ranks only containing *total* techniques. This is different from unit testing research and what Mostafa et al. (2017) assume. We see two reasons for this: (1) performance changes are captured by benchmarks that cover similar parts of the software under test; and (2) a large subset of the benchmarks in a suite have overlapping coverage with other benchmarks, where the average overlap is between 25% and 37% (see Table 3).

The best performing techniques (with rank 1) are the ones using *dynamic-coverage* and granularity *benchmark-parameter* in combination with the *total* strategy. The first *additional* techniques using *dynamic-coverage* achieve rank 4, again with benchmark granularity *benchmark-parameter*. For both *dynamic-coverage* strategies, the granularity *benchmark-method* performs worse compared to *benchmark-parameter*, indicating that they should prioritize benchmarks on parameter level to be more effective.

In terms of *static-coverage*, the best techniques achieve a higher rank than the first *additional* technique with *dynamic-coverage*, i.e., rank 2 and 3. Interestingly, all of these use the most imprecise static CG algorithm, i.e., *sc-algo*^RTA^. A reason could be that the more precise *sc-algo*^OCFA^ and *sc-algo*^O1CFA^ show a higher number of benchmarks without coverage information, i.e., 28% or higher as compared to 21% or lower for *sc-algo*^RTA^ (see Table 3). Reif et al. (2019) demonstrate that reflection is a common cause for unsoundness of static CGs, which potentially affects the higher percentage of empty coverage sets and, consequently, the lower effectiveness. Similar to *dynamic-coverage* techniques, *static-coverage* techniques with granularity *benchmark-parameter* tend to outperform techniques with *benchmark-method*.

Regarding the reflection option (*sc-ro*) or entrypoint set (*sc-ep*), we do not observe particular differences in *APFD-P*. For *sc-algo*^OCFA^ and *sc-algo*^O1CFA^, Table 3 shows that there is hardly any coverage difference between the reflection options *sc-ro*^NONE^ and *sc-ro*^MAX^ (when all other coverage-specific parameters are fixed). Although this is not the case for *sc-algo*^RTA^, *sc-ro* does not seem to have a big impact on *APFD-P*. The entry point set (*sc-ep*) for all CG algorithms (*sc-algo*) has an impact on the number of covered methods, *sc-ep*^*s*^ results in larger CGs per benchmark than *sc-ep*^*m*^, but no impact on the overlap, and only a minor impact on the empty coverage sets. Nonetheless, we do not see their impact on the coverage information reflected in the *APFD-P* effectiveness.

Finally, only 2 of 54 techniques do not statistically outperform a *random* ordering across our projects.

#### *Top-3*

##### Per Project

Table 5 shows per project the *Top-3* effectiveness across all versions and all 54 TCP techniques, similar to Table 4. Different from *APFD-P*, a lower *Top-3* value is better, i.e., fewer benchmarks are required to be executed to find the three largest performance changes. This is reflected in Table 5, where columns “Mean Min.” and “Conf. Int. Lower” are further left as “Mean Max.” and “Conf. Int. Upper”, respectively.

**Table 5:** *Top-3* of the 54 TCP techniques per project compared to a *random* ordering

Project	Mean		Conf. Int.		vs. *random*		
	Min.	Max.	Lower	Upper	***+***	***=***	***−***
*Byte Buddy*	0.57	0.73	0.48	0.81	40	7	7
*Eclipse Collections*	0.29	0.62	0.16	0.83	54	0	0
*JCTools*	0.44	0.73	0.27	0.85	53	0	1
*Jenetics*	0.53	0.67	0.43	0.78	46	8	0
*Log4j 2*	0.48	0.75	0.33	0.82	22	16	16
*Netty*	0.39	0.68	0.20	0.86	38	16	0
*Okio*	0.45	0.71	0.26	0.86	46	0	8
*RxJava*	0.51	0.68	0.37	0.79	48	6	0
*Xodus*	0.37	0.68	0.23	0.85	42	8	0
*Zipkin*	0.66	0.70	0.54	0.79	0	54	0

We observe that the range of mean *Top-3* values is wide, where depending on the project and technique between 16% (0.16) and 86% (0.86) of the full benchmark suite must be executed to capture the three largest performance changes. This shows that TCP can be effective regarding *Top-3* in the best cases, but it can also have almost no benefit over executing the full suites if the worst technique is utilized. Depending on the project, the best technique requires executing between 29% (*Eclipse Collections*) and 66% (*Zipkin*).

It is more often the case than for *APFD-P* that any TCP technique provides a benefit in terms of *Top-3* over a *random* ordering. For eight projects, i.e., *Byte Buddy*, *Eclipse Collections*, *JCTools*, *Jenetics*, *Netty*, *Okio*, *RxJava*, and *Xodus*, the majority of techniques are superior to *random*. For *Log4j 2*, more techniques are inferior or equal and, therefore, not effective compared to *random*. However, for this project 22 techniques are superior. An interesting project is *Zipkin*, for which *all* techniques perform equal to *random*. *Zipkin* is also the project that shows the lowest *APFD-P* values among all projects (see Table 4). Nonetheless, these results show that most TCP techniques enable capturing the largest performance changes early.

##### Overall

**Fig. 8:**
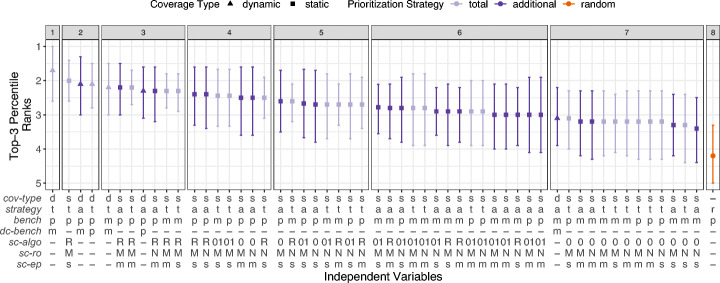
*Top-3* ranks across all projects and versions, and for a threshold *t* = 3*%*. For the independent variable descriptions, see Table 2

Similar to *APFD-P* and Fig. 7, Fig. 8 shows the results of the double Scott-Knott test (analysis step 3) across all projects.

In line with the *APFD-P* results, the sole, best TCP technique (rank 1) employs *dynamic-coverage* in combination with the *total* strategy, prioritizes benchmark parameterizations (*benchmark-parameter*), and retrieves coverage information only once per benchmark method (*dc*-*bench*^*m*^). The *additional* strategy is generally more effective than for *APFD-P*, with the first technique already ranked in cluster 2. However, almost all *additional* techniques are ranked one cluster lower than the corresponding *total* technique with the same parameters.

Regarding *static-coverage*, the best performing technique is the same as for *APFD-P*, i.e., *total* strategy with granularity *benchmark-parameter* constructing CGs with *sc-algo*^RTA^, *sc-ro*^MAX^, and *sc-ep*^*s*^. Techniques employing *sc-algo*^RTA^ again perform better than techniques with more precise CG analyses. The first technique using a different CG algorithm has rank 4. Techniques relying on *sc-algo*^RTA^ likely perform better due to significantly fewer top 3 benchmarks with empty coverage sets (see Table 3). Different from *APFD-P*, *sc-algo*^OCFA^ performs the worst, with all techniques but the ones using the *additional* strategy and *benchmark-parameter* having rank 7, only one above a *random* ordering. The best *static-coverage* techniques (rank 2 and 3) almost exclusively rely on the highest reflection option parameter available, i.e., *sc-ro*^MAX^. Nonetheless, *sc-ro* does not have a considerable impact on lower ranked *static-coverage* techniques. Finally, we do not observe a *Top-3* difference when using CGs with distinct entry points per benchmark (*sc-ep*^*m*^) or unified entry points across all benchmarks (*sc-ep*^*s*^).

For both, *static-coverage* and *dynamic-coverage* techniques, benchmark granularity *benchmark-parameter* performs better (or equal) than *benchmark-method*. This is in line with the findings from *APFD-P*. Overall, none of the 54 techniques perform worse than a *random* ordering. 
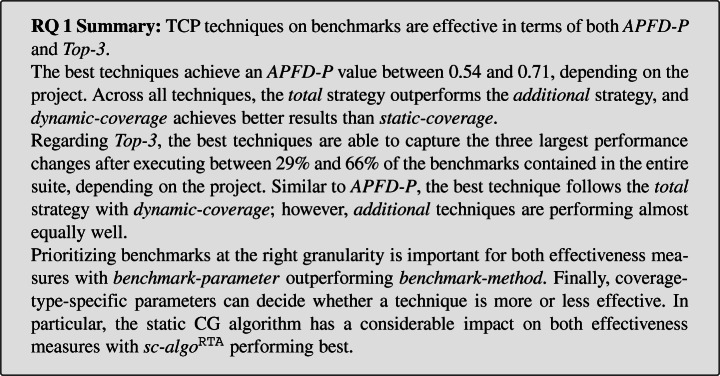


### RQ 2: Robustness

This section presents how robust the TCP techniques’ effectiveness is with respect to what magnitude of performance change is considered significant (see Section 4.4.1). To this end, we perform the following two analyses using a diverse set of thresholds *t* ∈ *T*, where *T* = {0,1,2,3,4,5,6,7,8,9,10,15,20,25,50,100}:

1. We investigate the impact of the different thresholds *t* on the overall ranking of TCP techniques.
2. We study the effectiveness difference of the techniques when using different thresholds *t*.

Note, we only investigate *APFD-P* robustness and refrain from analyzing *Top-3* robustness, as by construction *Top-3* considers the benchmarks exhibiting the 3 largest performance changes, which do not change for different performance change size thresholds *t*.

#### Technique Ranking across all Thresholds *t*

To investigate whether the overall effectiveness and the ranking among the 54 techniques change if we consider the *APFD-P* values of all studied thresholds *t*, we perform the analysis steps of RQ 1 (see Section 5.1) with the following minor modifications:

1. We calculate the *APFD-P* values for every threshold *t* individually. This results in a single *APFD-P* value for each TCP technique applied to a project, a version, and a threshold *t*.
2. We apply the Scott-Knott ESD test for every project, where a TCP technique is represented by the *APFD-P* values it achieves in every version for every threshold *t* as opposed to every version with a single threshold *t* (i.e., *t* = 3 for RQ 1). This provides us with a single rank per technique and project, considering all thresholds *t*.
3. We apply the Scott-Knott ESD test again on the ranks of the previous analysis step. This step remains unchanged compared to RQ 1.

**Fig. 9:**
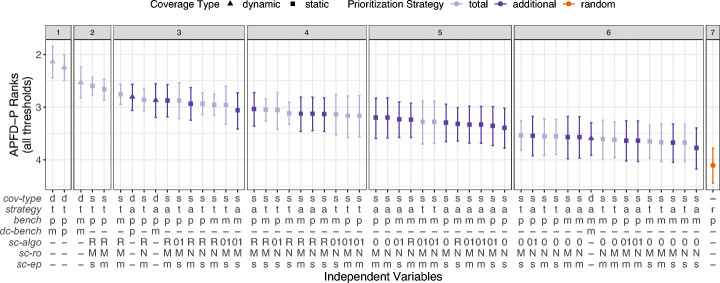
*APFD-P* ranks across all projects and versions, and for all thresholds *t*, where *t* ∈{0,1,2,3,4,5,6, 7,8,9,10,15,20,25,50,100}. For the independent variable descriptions, see Table 2

Figure 9 shows the *APFD-P* ranks for each TCP technique across all projects, versions, and thresholds. Similar to Fig. 7, techniques that have no statistical significant difference among each other are ranked in the same cluster (facet). Techniques that are further to the left perform better, and techniques that are further to the right perform worse.

The results are largely similar to the *APFD-P* results from RQ 1 in Fig. 7. The *total* techniques still perform the best, with the top 5 techniques (ranked 1 and 2) exclusively being *total* techniques. Similarly, techniques with *dynamic-coverage* outperform the ones with *static-coverage*. Nevertheless, we notice three differences where the threshold has a non-negligible impact on the ranking:

- Techniques with *additional* strategies “catch up” to *total* strategies, with the first one already having rank 3 as opposed to rank 4.
- All techniques now perform better than a *random* ordering, whereas two techniques (*additional* with *static-coverage*) performed equally to *random* when considering the specific threshold *t* = 3.
- The confidence intervals are considerably narrower, indicating that the techniques’ *APFD-P* ranks are more stable, which gives us high confidence in the robustness of the ranking, even if a different threshold *t* is chosen.

#### Effectiveness Variation across different Thresholds *t*

The previous section showed that different thresholds *t*, have a minor impact on the *APFD-P* rank of the TCP techniques. We now investigate whether the *APFD-P* value of a particular technique changes with different thresholds *t*. For this, we apply the following analysis steps:

1. Similar to analysis step (1) of RQ 1 (see Section 5.1), we first calculate *APFD-P* for every combination of the projects; their versions; the 54 TCP techniques; and, different from RQ 1, the different performance change size thresholds *t*. This results in a single *APFD-P* value for each combination.
2. We then calculate the *APFD-P* robustness for each TCP technique per project and version, as defined as the difference between the maximum and minimum *APFD-P* value. Intuitively, the robustness describes by how much the *APFD-P* values change when using different thresholds *t*. Let’s define the *APFD-P* value of a particular TCP technique *TCP*, for a project *p*, in a version *v*, and for a threshold *t* as $e^{TCP, p, v}_{t}$. The set of all effectiveness values is then $E^{TCP, p, v} = \bigcup _{t \in T} e^{TCP, p, v}_{t}$. Finally, the robustness *r*^*TCP*,*p*,*v*^ is then defined as *r*^*TCP*,*p*,*v*^ = *max*(*E*^*TCP*,*p*,*v*^) − *min*(*E*^*TCP*,*p*,*v*^), with *min* and *max* being the minimum and maximum *APFD-P* value *e*, respectively. A robustness value *r* of 0.0 means that a TCP technique is robust and does not change with different thresholds *t*, whereas a robustness value of 1.0 indicates a completely unstable technique with large effectiveness differences for different thresholds *t*.
3. Finally, we apply the Scott-Knott ESD test for the TCP techniques, combining the robustness values of all projects and versions, at significance level *α* = 0.05. This results in a single cluster rank per technique across all projects.

##### Per Project

**Fig. 10:**
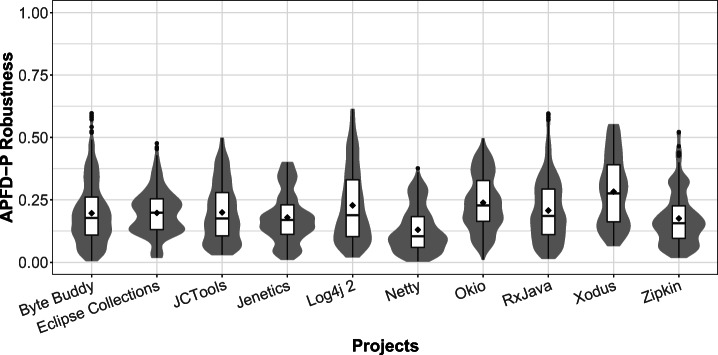
*APFD-P* robustness for each project across all versions and TCP techniques, when considering the thresholds *t* ∈{0,1,2,3,4,5,6,7,8,9,10,15,20,25,50,100}. The bar indicates the median, the diamond the mean, the box the IQR, and the whiskers [*Q*_1_|*Q*_3_] + 1.5 ∗ *IQR*

Figure 10 shows the *APFD-P* robustness (y-axis) for each project (x-axis), where each data point of a project (*p*) is a robustness value *r*^*TCP*,*p*,*v*^, for all TCP techniques (*TCP*) in all versions (*v*).

We observe that the threshold *t* has a considerable impact on a technique’s *APFD-P* value. Depending on the project, technique, and version, the *APFD-P* values vary between 0 and 0.62. *Netty* is the least-impacted with a median robustness of 0.11, whereas *Xodus* is the most impacted project with 0.28. This shows that the decision of what is a significant performance change has a drastic impact on the evaluation of TCP techniques.

##### Overall

**Fig. 11:**
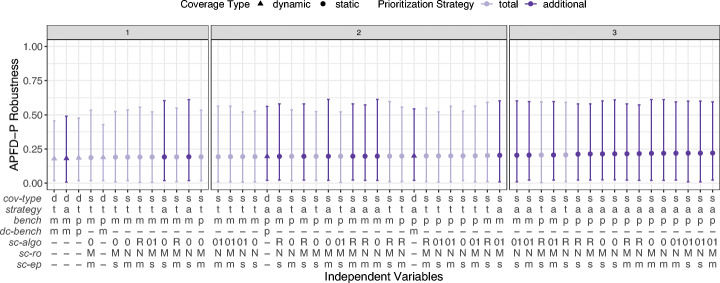
*APFD-P* robustness for each TCP technique across all projects and versions, when considering the thresholds *t* ∈{0,1,2,3,4,5,6,7,8,9,10,15,20,25,50,100}. The shapes indicate the mean, and the whiskers show the minimum and maximum. For the independent variable descriptions, see Table 2

Figure 11 depicts the *APFD-P* robustness (y-axis) for each TCP technique, across all projects and versions. Techniques to the left are more robust than ones to the right, also indicated by the Scott-Knott rank (facets) reported by analysis step (3). Note that whiskers represent minimum and maximum robustness values and not confidence interval bounds as in previous figures.

We observe that although there are statistically significant differences between the three clusters, the mean robustness does not change much among the techniques. The *total* techniques tend to be more robust than the *additional* techniques, as the majority of techniques with rank 1 use the *total* strategy, and the majority of techniques with rank 3 use the *additional* strategy. 4 of 6 techniques with *dynamic-coverage* are ranked 1, whereas the other two are ranked 2. In terms of benchmark granularity, techniques with *benchmark-method* tend to be more robust than ones with *benchmark-parameter*. Finally, we do not observe robustness differences between techniques with different coverage-type-specific parameters, i.e., neither for techniques with *dynamic-coverage* (*dc-bench*) nor for techniques with *static-coverage* (*sc-algo*, *sc-ro*, and *sc-ep*). 
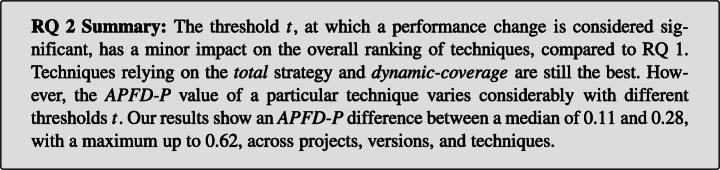


### RQ 3: Efficiency

**Fig. 12:**
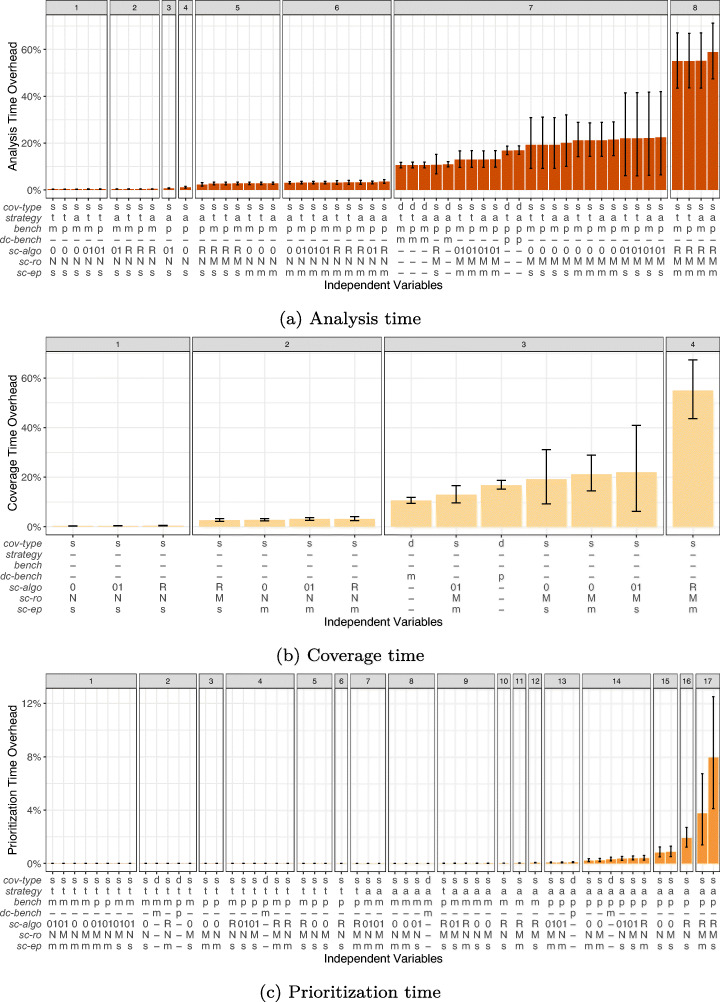
Efficiency of techniques in terms of analysis time (i.e., coverage and prioritization time) in runtime overhead in percent of the full benchmark suite execution. Error bars represent the 95% confidence interval. For the independent variable descriptions, see Table 2

This section presents the efficiency of the studied TCP techniques, as defined by their runtime overhead with respect to a full benchmark suite execution. Figure 12 presents the three efficiencies across all projects and versions: (1) analysis time, which is the sum of the following two times, in Fig. 12a; (2) coverage time, i.e., the time it takes to extract coverage information, in Fig. 12b; and (3) prioritization time, i.e., the time it takes to prioritize all benchmarks of a suite based on the coverage information, in Fig. 12c. The techniques are ranked from the lowest overhead on the left to the highest overhead on the right and are, again, clustered into ranks with the Scott-Knott ESD test (similar to RQ 1 and RQ 2). The y-axis depicts the mean runtime overhead for each technique across all projects and versions. Individual technique runtimes, i.e., of a particular project in a specified version, are normalized by the execution time of the full benchmark suite of the particular project and version. Whiskers represent the 95% bootstrap confidence interval of the mean overhead.

#### Analysis Time

From Fig. 12a, we observe that the 27 techniques ranked in clusters 1 to 6 have a range of mean overheads between < 1% and 3.7%, with confidence interval bounds from < 1% to 4.5%. These techniques exclusively use *static-coverage*, with the majority relying on *sc-algo*^OCFA^ and *sc-algo*^O1CFA^. All techniques use the lowest reflection option (*sc-ro*^NONE^) and/or use a single CG entry point set (*sc-ep*^*s*^).

The 23 techniques ranked in cluster 7 include all *dynamic-coverage* and *static-coverage* techniques relying on the algorithms *sc-algo*^OCFA^ and *sc-algo*^O1CFA^ in combination with multiple entry point sets (one per benchmark; *sc-ep*^*m*^). This cluster contains the techniques with the widest variety of mean overheads, ranging from 10% to 23%, with confidence interval bounds between 6% and 42%.

The *dynamic-coverage* techniques show a low overhead variability among the different projects and versions, with mean overheads between 10% and 17%. Although all *dynamic-coverage* techniques are ranked in cluster 7, there is a significant difference between techniques with *dc*-*bench*^*m*^ (10.7%) and *dc*-*bench*^*p*^(16.8%).

The techniques with the highest runtime overhead rely on *static-coverage* in combination with *sc-algo*^RTA^, *sc-ro*^MAX^, and *sc-ep*^*m*^. Their mean overhead ranges from 55% to 59%, with confidence interval bounds between 43% and 72%.

Finally, we neither observe differences in analysis time overhead between *total* and *additional* strategies nor *benchmark-method* and *benchmark-parameter* granularities. This indicates that prioritization time contributes less to the analysis time than coverage time.

#### Coverage Time

Figure 12b shows the coverage time overhead per combination of coverage-type-specific parameters. This figure is different from all the others because (1) coverage extraction is the first step of the analysis performed by a TCP technique and, hence, it can not be affected by previous steps; and (2) different prioritization strategies and benchmark granularities rely on the same coverage information.

We observe that the coverage time has similar overhead numbers as the analysis time, and they are ranked into four clusters. This is a first indication that coverage time is indeed the deciding factor for a TCP technique’s analysis time.

Coverage extractors with rank 1 are using the lowest reflection option (*sc-ro*^NONE^), a single CG entry point set (*sc-ep*^*s*^), and only differ by their CG algorithm (*sc-algo*^RTA^, *sc-algo*^OCFA^, and *sc-algo*^O1CFA^). All these extractors have an overhead below 1%.

The second rank (2), again, contains only *static-coverage* extractors. Three of these are the same extractors as in cluster 1 but with entry point sets per benchmark (*sc-ep*^*m*^). The remaining extractor with rank 2 employs *sc-algo*^RTA^ with the maximum reflection option (*sc-ro*^MAX^) and a single entry point set (*sc-ep*^*s*^). Their mean overhead ranges from 2.7% to 3.2%.

Cluster 3 contains all *dynamic-coverage* extractors and all remaining but one *static-coverage* extractor (which we discuss below). Their mean overheads are between 10.6% and 22.1%, with confidence interval bounds ranging from 6% to 41%. Interestingly, these extractors make up all but one of the TCP techniques in analysis time cluster 7 (see Fig. 12a). The overheads of the *dynamic-coverage* extractors are responsible for the majority of the analysis time of the dependent TCP techniques.

Finally, the coverage extractor with the highest overhead (ranked 4) retrieves *static-coverage* using *sc-algo*^RTA^ in combination with *sc-ro*^MAX^ and *sc-ep*^*m*^. This also explains the worst TCP techniques (in analysis time cluster 8) that all rely on this extractor.

The overheads from the extractors in cluster 3 and 4 are almost equal to the TCP techniques’ analysis times in clusters 7 and 8. This shows that coverage is the major factor of long analysis times of TCP techniques.

#### Prioritization Time

Figure 12c shows the prioritization time overhead per TCP technique across all projects and versions. Note that here we are again interested in all 54 TCP techniques.

We first observe that the majority of the techniques, i.e., 51 of 54, have a mean prioritization time overhead below 1%. This confirms the suggested finding that coverage time and *not* prioritization time is the main contributor to TCP efficiency, for most of the studied techniques. Nevertheless, three techniques show overheads worth mentioning (ranked 16 and 17); all three rely on *static-coverage*, apply the *additional* strategy with granularity *benchmark-parameter*, and use *sc-algo*^RTA^ as CG algorithm.

The technique in rank 16 uses the lowest reflection option (*sc-ro*^NONE^) in combination with a single CG entry point set (*sc-ep*^*s*^), resulting in a mean overhead of 2%, with confidence interval bounds between 1.2% and 2.8%. The reason why this technique is only one cluster away from the worst techniques is because of the high number of covered methods per benchmark, i.e., on average 3,005.32 as depicted in Table 3. However, this technique has a relatively low analysis time overhead of 2.3%, which is largely caused by the prioritization overhead.

Finally, the two techniques with the highest overhead (with rank 17) use the maximum reflection option (*sc-ro*^MAX^). Their mean overhead is 3.8% and 8% for *sc-ep*^*m*^ and *sc-ep*^*s*^, respectively. The technique with *sc-ep*^*m*^ also has the highest mean analysis time overhead at 59%; and the technique with *sc-ep*^*s*^ is the only one in analysis time cluster 7, which is due to the high prioritization overhead. Both techniques owe their high prioritization overheads to the number of covered methods per benchmark, i.e., on average 7,497.67 (*sc-ep*^*m*^) and 14,855.13 (*sc-ep*^*s*^). 
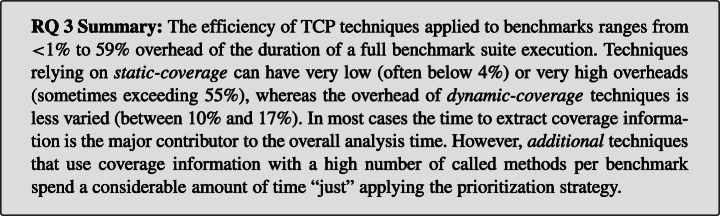


## Discussion and Recommendations

In this section, we discuss the trade-off between TCP effectiveness and efficiency, provide recommendations for researchers and practitioners, and compare our findings to the findings of TCP for unit tests.

### Choosing a TCP Technique

Our results, especially RQ 1 and RQ 3, show that the studied TCP techniques are to a varying degree effective and efficient. However, whether a specific technique is also useful in practice depends on both effectiveness and efficiency.

We have found that the most effective techniques in terms of *APFD-P* and *Top-3* use *dynamic-coverage*. The best *dynamic-coverage* technique uses the *total* strategy, *benchmark-parameter*, and *dc*-*bench*^*m*^ and has an analysis time overhead of approximately 11%. In case of very long-running benchmark suites (e.g., 2.71h for *Log4j 2* or 38.45h for *Eclipse Collections*), even a 11% overhead may still be worthwhile if it leads to large performance changes being identified sooner.

However, if an 11% overhead is too expensive, a technique relying on *static-coverage* might be an attractive alternative. The most effective *static-coverage* technique, for both *APFD-P* and *Top-3*, in our study uses the *total* strategy, *benchmark-parameter*, *sc-algo*^RTA^, *sc-ro*^MAX^, and *sc-ep*^*s*^. This technique is also efficient with a mean analysis overhead of below 3%.

It is important to keep in mind that TCP can be less effective than a *random* ordering, depending on the project and the parameterization of the technique (see Tables 4 and 5). However, on average across all studied projects all techniques are superior to *random* (see Figs. 7, 8 and 9).

Practitioners who are keen on applying TCP for their microbenchmark suites should carefully evaluate whether they would benefit from it, by answering the following questions:

1. Is the suite runtime too long to wait for its completion, and can we, therefore, benefit from prioritization?
2. Which analysis overhead is acceptable (in relation to the suite runtime)?
3. Which technique is effective *and* efficient for our project?

### Efficiency of Static vs. Dynamic Coverage Techniques

A few *static-coverage* techniques are almost as effective as *dynamic-coverage* techniques, and the majority are as efficient or considerably more efficient than *dynamic-coverage* techniques. However, *static-coverage* is always faster than *dynamic-coverage* can not be generally assumed, i.e., extensive overheads are possible with the “wrong” technique parameterization.

Our results show that in most cases the coverage time is the deciding factor whether a technique is fast or slow. For example, highly effective *static-coverage* techniques, such as the ones ranked in cluster 3 for *APFD-P* (see Fig. 7) as well as *Top-3* (see Fig. 8), have the highest runtime overhead at above 50%. Often a change of one coverage-type-specific parameter can already improve the efficiency drastically without sacrificing effectiveness. The technique relying on the *total* strategy with *static-coverage*, *benchmark-parameter*, *sc-algo*^RTA^, and *sc-ep*^*m*^ is equally effective for either *sc-ro* value. However, *sc-ro*^MAX^ has a mean overhead of 55%, whereas for *sc-ro*^NONE^ the overhead is below 3%. This shows that sophisticated reflection handling mechanisms of static CG libraries can have a detrimental impact on the effectiveness of *static-coverage* techniques.

### Impact of Coverage-Type-Specific Parameters

Our study is, to the best of our knowledge, the first to define coverage-type-specific parameters of TCP techniques and to assess their impact on effectiveness and efficiency. Previous studies either fixed these parameters, e.g., the static CG algorithm, or do not explicitly mention them (Zhang et al. 2009b; Luo et al. 2016; Luo et al. 2019; Mostafa et al. 2017). Our results show that these parameters can have an impact on both effectiveness and efficiency. Hence, they cannot be neglected in rigorous experimental evaluations. We hypothesize that there is a similar impact of coverage-type-specific parameters on TCP effectiveness in functional testing research. Future studies should validate this hypothesis.

For *dynamic-coverage* techniques, choosing between *dc*-*bench*^*m*^ and *dc*-*bench*^*p*^ can affect both effectiveness and efficiency: (1) favoring *dc*-*bench*^*m*^ over *dc*-*bench*^*p*^, i.e., retrieving *dynamic-coverage* per benchmark method rather than per benchmark parameterization, reduces the overhead from 17% to 11%; (2) while being more effective regarding *Top-3* effectiveness; and (3) remaining equally effective in terms of *APFD-P*.

For techniques with *static-coverage*, both effectiveness and efficiency is drastically impacted by coverage-type-specific parameters. Effectiveness changes mostly with different CG algorithms. Surprisingly, the least precise algorithm among the studied ones, i.e., *sc-algo*^RTA^, enables the most effective techniques. This is likely due to *sc-algo*^OCFA^ and *sc-algo*^O1CFA^ being not able to extract coverage information for many benchmarks (see Table 3). As already discussed before, changes to coverage-type-specific parameters can also lead to efficiency drops. Especially, more sophisticated reflection options, i.e., *sc-ro*^MAX^ instead of *sc-ro*^NONE^, and constructing CGs per benchmark with smaller, more specific entry point sets (*sc-ep*^*m*^), is often much less efficient at a similar effectiveness.

### Choice of Threshold

Our study’s robustness results (RQ 2 in Section 5.2) show that depending on which performance change size is considered to be significant, i.e., as defined by the threshold *t*, the concrete *APFD-P* values change on median between 0.11 and 0.28, depending on the project. Nonetheless, the technique rankings hardly change, which demonstrates that all techniques are similarly affected by different thresholds *t*.

One could argue that the threshold should always be set to *t* = 0, which would consider all performance change of any size for *APFD-P* calculation. However, this can be problematic for two reasons:

1. Performance experiments are prone to measurement bias, where the measurement result does not accurately reflect the (software) systems’ true performance (Georges et al. 2007; Mytkowicz et al. 2009; Curtsinger and Berger 2013). Non-obvious execution environment peculiarities can affect the measurement, such as environment variables, stack sizes, background processes, or frequency scaling. Consequently, a measured performance change might in fact be due to a confounding factor and not due to a change to the software. Even if one follows a rigorous measurement methodology, the absence of measurement bias can not be guaranteed. Therefore, false-positives in the detection of performance changes impact the effectiveness evaluation of TCP techniques. To manage measurement bias, a threshold *t* can filter out these changes.
2. Multiple co-occurring performance changes between two software versions are common (Chen and Shang 2017), but often they are of small size. Consequently, developers might only be interested in changes of a certain size, e.g., everything below a 10% change is not worth investigating. The exact threshold *t* that is relevant depends on the project, developer, and application scenario. Our results show that defining this threshold can considerably change the evaluated effectiveness of a technique.

We suggest that researchers conducting all kinds of performance experiments to consider different thresholds *t* when evaluating the effectiveness of their approaches. In particular, research on TCP for benchmarks must consider the sensitivity of the evaluation metric *APFD-P*. Practitioners eager to apply TCP on their benchmark suites should decide which performance change sizes they are interested in capturing early, as it can change which technique is optimal for their usage scenario.

### Comparison to TCP for Unit Tests

To assess how TCP for benchmarks compares to TCP for unit tests, we compare our results to the ones of Luo et al. (2019). Their study is the most recent, large-scale study of static and dynamic TCP techniques for unit tests.

Different from their study, ours investigates benchmark granularities on method (*benchmark-method*) and parameter (*benchmark-parameter*), whereas theirs looks at class and method level. The implementation of our *static-coverage* techniques resembles theirs, both are based on Zhang et al. (2009b). Our *dynamic-coverage* techniques rely on coverage information on *method-level*, whereas theirs rely on *statement-level* coverage.

There is a conceptual difference between dynamic TCP for unit tests and dynamic TCP for benchmarks: coverage information (for unit tests) is usually acquired during the test executions of the previous version. Luo et al. (2019) refrain from studying the efficiency of dynamic techniques, because “the temporal overhead is quite high and well-studied”. As benchmarks are executed many times to retrieve reliable results (see Section 2), TCP for benchmarks can utilize a single benchmark execution of a new version to instrument the code and retrieve dynamic coverage information, as described in Section 3.

Luo et al. (2019) report the following APFD values for TCP with method granularity (which is more effective than class granularity): techniques with *static-coverage* achieve on average 0.764 and 0.818 across their study objects, whereas the ones with *dynamic-coverage* reach 0.809 and 0.898, respectively for *total* and *additional* strategies.

**Fig. 13:**
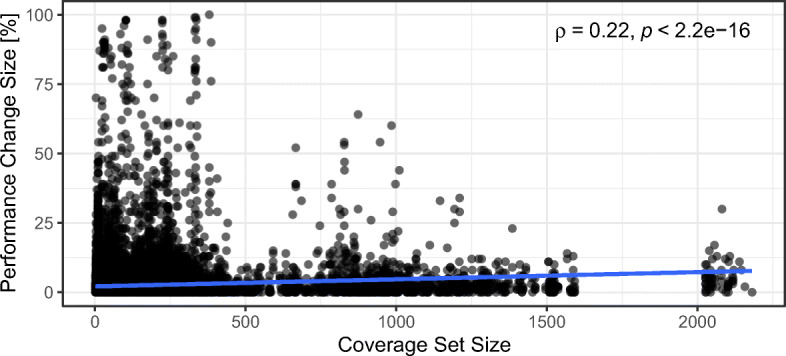
Relation between coverage set size and performance change size. Each dot represents a benchmark parameterization between two versions. The blue line depicts a linear model, and in the top right corner is the Spearman’s rank correlation coefficient *ρ* and *p*-value

Our results highlight four major observations compared to unit tests:

1. TCP is considerably less effective for benchmarks than for unit tests, if we assume that values for APFD and *APFD-P* are comparable. This is likely due to performance changes being less correlated with the number of covered methods (or statements) than functional faults are. Figure 13 depicts the relation between coverage set size and performance change size. The Spearman’s rank correlation test validates that there is only a low correlation at *ρ* = 0.22. To circumvent this situation, TCP for benchmarks requires better approximations for performance changes than “just” the sum of all covered items. To this end, Mostafa et al. (2017) build a performance change impact model for collection-intensive software, and Chen et al. (2020) build a runtime-cost-aware machine learning model. However, both studies (partially) evaluated their techniques with unit test suites which are executed in a benchmark-like fashion. In this study, we explored the state of traditional TCP applied to benchmarks among a large set of parameter combinations. It is our hope that future research can use this foundation to develop techniques that are more effective for prioritizing benchmarks.
2. The *total* strategy is more effective than the *additional* strategy for benchmarks when relying on either *static-coverage* or *dynamic-coverage*; whereas the opposite is true for TCP for unit tests. A potential reason for this relates to the definition of *APFD-P* by Mostafa et al. (2017): it does not distinguish between multiple (performance) faults detected by the same benchmark as APFD does, but it considers benchmark results as a single fault with different severities, i.e., the performance change size. Future research should aim at devising a new effectiveness metric for TCP for benchmarks. This would require building a data set that distinguishes root causes of distinct performance changes (with their severities/change sizes) per benchmark. However, it is unclear whether this is feasible, as performance is non-linear and not directly additive (Siegmund et al. 2015).
3. The efficiency of TCP for benchmarks is less of a concern compared to TCP for unit tests, at least for the majority of the studied techniques. About half of the *static-coverage* techniques have an overhead below 4%, whereas the *dynamic-coverage* techniques have an overhead between 10% and 17%. This reasonable overhead potentially makes TCP for benchmarks applicable in practice.
4. The performance change of a benchmark executed on two adjacent versions is a continuous, open-ended value, whereas the outcome of a unit test is binary, i.e., either it exposes a fault or not (disregarding flaky tests for simplicity). This leads to the challenge that measurement uncertainty and bias impacts the robustness of the technique evaluation, as studied for RQ 2 in Section 5.2.

### What is an Important Performance Change?

This paper’s goal is to reorder microbenchmarks in a suite to detect more important performance changes sooner. For this, we define the importance of a performance change to be linked to the performance change size: a larger performance change is more important than a smaller performance change, as proposed by Mostafa et al. (2017). While it may seem natural to use this definition, developers might perceive different benchmarks as more important than the ones that exhibit the largest change. The definition is central to the effectiveness of the TCP techniques, and other definitions are likely to lead to different results and conclusions.

It is not clear how performance changes of benchmarks translate to overall end-to-end-performance of a software system. A developer might be more interested in benchmark changes that contribute more to the overall performance. This, however, is non-trivial to assess:

1. This paper’s projects under investigation all fall into the category of software libraries. It is unclear what the end-to-end performance of libraries is. They have multiple (API) endpoints and might be used differently by different developers.
2. An application or system benchmark could be the gold standard for important performance changes. For example, such benchmarks exist for the JVM, such as SPECjvm (Standard Performance Evaluation Corporation (SPEC) 2008), DaCapo (Blackburn et al. 2006), Da Capo con Scala (Sewe et al. 2011), and Renaissance (Prokopec et al. 2019). Moreover, Grambow et al. (2021) recently employed application benchmark traces to improve microbenchmark suites. However, it is unclear how to map from microbenchmark changes to application benchmark changes.

One might also define the importance of microbenchmarks based on whether they cover parts of the library that is important to API clients. Following this idea, a large performance change in a hardly used part is probably less important than a small performance change in a heavily used part. One could get this importance definition by either (1) dynamically tracing unit test executions of API clients, similar to Laaber and Leitner (2018); or (2) statically mining large software repositories, e.g., with a technique as proposed by Sawant and Bacchelli (2017).

Our definition treats different change sizes as differently important. However, developers might deem any detected performance change, irrespective of the size, as important, as long as it is above a certain threshold. Such a definition would be close to how unit testing research treats functional faults, i.e., a test or benchmark either fails or succeeds.

Moreover, Mostafa et al. (2017)’s and our effectiveness definition, i.e., *APFD-P* and *Top-3*, treats regressions and improvements the same way: they are performance changes. In a regression testing scenario, such as the one in this paper, developers might care more (if not only) about performance regressions and are less concerned with performance improvements. In this case, TCP techniques should prioritize benchmarks that potentially expose performance regression over ones that do not exhibit changes or only report improvements. This preference of performance regressions should also be reflected in the effectiveness metrics.

Finally, which benchmarks or performance changes are really important for developers can ultimately only be answered by developers themselves. This, however, would be a study on its own, nonetheless an important one. Not only in the context of this study but more generally, future research in performance engineering/testing should involve developers more.

## Related Work

Our study is related to three main areas of research: (1) TCP of functional tests, (2) performance testing, and (3) performance measurements.

### Test Case Prioritization of Functional Tests

Regression testing for functional/unit tests has been extensively explored (Yoo and Harman 2012), with the three main techniques being test suite minimization, regression test selection (RTS), and test case prioritization (TCP). Our study takes the traditional TCP techniques on unit tests (Rothermel et al. 1999), i.e., *total* and *additional* strategies, and studies them in the context of software benchmarks.

TCP’s main idea is to reorder test cases with the goal of maximizing fault-exposure rate, i.e., finding more faults sooner. Rothermel et al. (1999, 2001) coined the term TCP and introduced the main techniques: *total* and *additional* strategies. Both are greedy, white-box prioritization techniques relying on coverage information, such as statement, branch, or method coverage. Where the *total* strategy assigns weights once to all tests and ranks them accordingly, the *additional* strategy re-assigns weights to prioritize tests that execute more, yet uncovered regions of the production code. Elbaum et al. (2002) extended the study to a total of 18 different techniques by rankings based on fault exposure and fault existence probabilities. Elbaum et al. (2001) extended APFD to incorporate cost of tests and faults.

More recent trends in greedy TCP techniques combine *total* and *additional* strategies (Zhang et al. 2013; Hao et al. 2014) or utilize less-expensive static coverage information (Zhang et al. 2009b; Mei et al. 2012). Other, non-greedy techniques have been proposed to utilize search-based algorithms (Walcott et al. 2006; Li et al. 2007), ant-colony optimization (Singh et al. 2010), knapsack solvers (Alspaugh et al. 2007), and integer linear programming (Zhang et al. 2009a). Time-aware techniques (Walcott et al. 2006; Alspaugh et al. 2007; Zhang et al. 2009a; Do et al. 2010; You et al. 2011) study the impact of time on TCP effectiveness.

With the emergence of CI and new code versions arriving at high velocity, efficient black-box techniques are on the rise (Elbaum et al. 2014; Liang et al. 2018; Haghighatkhah et al. 2018). Henard et al. (2016) investigate the differences between white-box and black-box techniques, and Luo et al. (2016, 2019) compare static and dynamic techniques.

Finally, recent efforts assess TCP techniques in real world contexts (Lu et al. 2016), contrast real faults to faults based on mutation (Luo et al. 2018), and incorporate developer knowledge into the ranking (Tonella et al. 2006).

Our study draws inspiration from many of the aforementioned papers. It studies traditional techniques, i.e., *total* and *additional* strategies, on method-level granularity, investigates the impact of varying prioritization parameters, focuses on efficiency, and applies all of it to software microbenchmarks.

### Performance Testing

Software performance engineering (SPE) can be conducted in two general ways: measurement-based and model-based (Woodside et al. 2007). Our work focuses on a specific technique of measurement-based SPE, i.e., performance testing with software microbenchmarks.

Traditional performance testing research dedicated their effort on system-level load testing, and the related stress, soak, and spike testing (Weyuker and Vokolos 2000; Menascé 2002; Jiang and Hassan 2015). More recent works in load testing focus on industrial contexts (Nguyen et al. 2014; Foo et al. 2015) and time reduction techniques (AlGhamdi et al. 2016; AlGhamdi et al. 2020; He et al. 2019).

The other form of performance testing, i.e., software microbenchmarking, has only received more attention from research in recent years. Software microbenchmarking is to load testing what unit testing is to functional system/integration testing. General studies empirically investigate the current state of software microbenchmarking (Leitner and Bezemer 2017; Stefan et al. 2017). Targeted research on their usage for raising the performance awareness of developers (Horký et al. 2015), the changes that they detect (Chen and Shang 2017), their applicability in CI (Laaber and Leitner 2018) shows the potential, but also the challenges, of using software microbenchmarks.

Challenges include the complexity of writing good microbenchmarks, executing them in a rigorous fashion, and assessing their results with statistical techniques. Damasceno Costa et al. (2019) devise a technique to statically detect bad practices, Laaber et al. (2019) study their behavior when executed on cloud infrastructure, and Bulej et al. (2012, 2017) introduce a declarative method for comparing different benchmark results using rigorous statistical testing. Ding et al. (2020) study whether unit tests can be effectively used for detecting performance changes. Laaber et al. (2020) devise an approach to stop microbenchmarks once their results are sufficiently stable; and Laaber et al. (2021a) employ machine-learning-based classifiers to predict whether a benchmark will be stable, based on statically-computed source code features, without the need to execute it.

In the context of regression testing, only a handful of studies have been conducted so far. Huang et al. (2014) predict the performance impact of a new software version to decide whether this new versions should be tested for performance. Pradel et al. (2014) and Yu and Pradel (2017) address performance regression testing for concurrent classes. Three regression test selection (RTS) techniques employ performance-impact prediction (de Oliveira et al. 2017), genetic algorithms (Alshoaibi et al. 2019), and machine learning classifiers (Chen et al. 2020) to select important benchmarks, i.e., the ones that are likely to expose performance changes, for every software version.

Closest to our work are the ones by Mostafa et al. (2017) and Chen et al. (2020), which are, to the best of our knowledge, the only other works on TCP for performance tests. Mostafa et al. (2017) focus on collection-intensive software and decide, based on code changes and a performance-impact model, which performance tests to prioritize. Their paper utilizes as baselines the “best techniques” based on unit testing research. We, however, outline that the assumption that TCP techniques from unit testing research behave identical for performance tests does not hold. The primary goal of Chen et al. (2020) is to predict whether tests are performance-affected, e.g., for RTS, but they also prioritize tests based on whether they are affected normalized by their runtime cost. Both works, however, (partially) use unit tests executed in a benchmark-like fashion as performance tests. It is unclear whether they are even comparable to dedicated performance tests, i.e., microbenchmarks, which are the objects in our study. We further show how the uncertainty of performance measurements and the choice of prioritization parameters impacts TCP effectiveness and efficiency.

### Performance Measurements

The results of any software benchmarking study are affected by the validity of the underlying performance measurements. A lot can go wrong, and many mistakes can be made. Consequently, measurement bias has in the past lead researchers to draw wrong conclusions (Mytkowicz et al. 2009). Effects due to memory layout (Curtsinger and Berger 2013) and dynamic compilation (Kalibera and Jones 2012; 2013) require careful experiment design and statistical evaluation. Georges et al. (2007) provide a guide for performance evaluations in Java. To retrieve reliable results from unreliable environments (such as clouds), Papadopoulos et al. (2019) outline a comprehensive methodology. We follow the methodologies from Georges et al. (2007) for the performance changes used in the effectiveness measure calculation, apply cloud performance measurement methodologies (Papadopoulos et al. 2019; Laaber et al. 2019) for the efficiency results, and employ rigorous statistical techniques (Kalibera and Jones 2012).

## Conclusions

This paper presents the first investigation on whether standard TCP techniques from unit testing research are applicable in the context of software microbenchmarks. We empirically studied the effectiveness, robustness, and efficiency of these techniques and investigated the impact of four independent variables, i.e., *total* and *additional* strategies, benchmark granularities on *method* and *parameter* level, *dynamic* and *static* coverage types, and four coverage-type-specific parameters. The unique combinations of these independent variables results in 54 different TCP techniques, which we evaluated on a large *JMH* data set comprising 10 Java OSS projects, across 161 versions, having 1,829 distinct microbenchmarks with 6,460 distinct parameterizations.

We found that techniques with the *total* strategy outperform *additional* techniques. The mean effectiveness ranges between 0.54 and 0.71 *APFD-P*, and it requires executing between 29% and 66% (*Top-3*) of the total benchmark suite. The performance change size, which is considered to be significant, impacts the effectiveness and can change the *APFD-P* values considerably, i.e., by a median difference of between 0.11 and 0.28. However, the ranking among different techniques is hardly affected by it. In terms of efficiency, we showed that the best technique has an overhead of 11% and uses *dynamic-coverage*, making TCP for benchmarks feasible. Techniques with *static-coverage* often reduce the overhead even further, often below 4%, while still being competitive in terms of effectiveness. Our efficiency analysis also revealed that the assumption that *static-coverage* is always cheaper than *dynamic-coverage* does not hold; “wrong” parameterization can drastically decrease efficiency, sometimes exceeding 55% of overhead. The choice of independent variable values has a considerable effect on effectiveness and efficiency, sometimes even rendering the TCP technique inferior to a *random* ordering and imposing a large analysis overhead.

Our results are of high importance to future research that considers standard TCP techniques as baselines for novel techniques, and they raise awareness of how impactful prioritization parameters are on TCP effectiveness and efficiency.

### Future Research

It is our hope that this paper is only the beginning of performance test prioritization. We envision five directions that seem worthwhile investigating.

- Current TCP techniques for performance tests are either tailored to specific types of software (Pradel et al. 2014; Mostafa et al. 2017) or evaluated on unit tests which are used as performance tests (Chen et al. 2020). Future research should devise generally applicable TCP techniques for benchmarks and evaluate these and existing techniques on benchmarks.
- This study investigated white-box TCP techniques, whereas black-box techniques have not been explored. These could be based on benchmark quality attributes or test similarity.
- It is unclear which information developers need to decide which benchmarks to execute on new commits and in which order. Empirical studies involving humans could influence design decisions for better benchmark TCP and RTS.
- Choosing TCP hyper-parameters (i.e., independent variable values) to maximize TCP effectiveness and efficiency is non-trivial, which would require better support for developers.
- Combining TCP and RTS techniques might provide optimal results in temporally-constrained settings, such as CI.

## Footnotes

1 https://junit.org/junit5

2 https://junit.org/junit5/docs/current/user-guide/#writing-tests-parameterized-tests

3 https://hg.openjdk.java.net/code-tools/jmh/rev/b45d214529fc

4 https://asm.ow2.io

5 https://github.com/wala/WALA

6 https://www.jacoco.org

7 all defined in rx.operators.RedoPerf

8 io.netty.handler.codec.DateFormatter2Benchmark.parseHttpHeaderDate FormatterNew
